# Serum hepcidin level, iron metabolism and osteoporosis in patients with rheumatoid arthritis

**DOI:** 10.1038/s41598-020-66945-3

**Published:** 2020-06-18

**Authors:** Hiroe Sato, Chinatsu Takai, Junichiro James Kazama, Ayako Wakamatsu, Eriko Hasegawa, Daisuke Kobayashi, Naoki Kondo, Takeshi Nakatsue, Asami Abe, Satoshi Ito, Hajime Ishikawa, Takeshi Kuroda, Yoshiki Suzuki, Ichiei Narita

**Affiliations:** 1 Niigata University Health Administration Center, 2-8050 Ikarashi, Nishi-ku, Niigata City, 950-2181 Japan; 2 Division of Clinical Nephrology and Rheumatology, Niigata University Graduate School of Medical and Dental Sciences, 1-757 Asahimachi-Dori, Chuo-ku, Niigata City, 951-8510 Japan; 3 Department of Rheumatology, Niigata Rheumatic Center, 1-2-8 Honcho, Shibata City, 957-0054 Japan; 4 Department of Nephrology and Hypertension, Fukushima Medical University, 960-1295, 1 Hikariga-oka, Fukushima City, Japan; 5 Division of Orthopedic Surgery, Niigata University Graduate School of Medical and Dental Sciences, 1-757 Asahimachi-Dori, Chuo-ku, Niigata City, 951-8510 Japan

**Keywords:** Biomarkers, Rheumatology

## Abstract

Hepcidin, a major regulator of iron metabolism and homeostasis, is regulated by inflammation. Recent studies have suggested that hepcidin and iron metabolism are involved in osteoporosis, and the aim of this study was to determine whether serum hepcidin levels are correlated with the degree of osteoporosis in patients with rheumatoid arthritis (RA). A total of 262 patients with RA (67.5 ± 11.4 years; 77.5% female) were enrolled. Serum iron, ferritin, and hepcidin levels were positively correlated each other. Multiple regression analyses revealed that the serum iron level was positively correlated with femoral T and Z scores, whereas the serum hepcidin level was not. Serum hepcidin level was correlated with the serum 25-hydroxy vitamin D level, which was in turn positively related to the femoral Z score. Serum hepcidin and serum iron were indirectly and directly related to osteoporosis in patients with RA.

## Introduction

Rheumatoid arthritis (RA), a risk factor for osteoporosis, is accompanied by both periarticular and systemic osteoporosis. The mechanisms of osteoporosis associated with RA involve the influence of inflammatory cytokines, such as interleukin (IL)-1, IL-6, and tumor necrosis factor (TNF); glucocorticoid treatment; and immobility.

Fibroblast growth factor 23 (FGF23) is bone-derived hormone for phosphorus diuresis that inhibits phosphate reabsorption and active vitamin D synthesis in the kidney. When kidney function decreases, the serum FGF23 level increases^1^. Previously we reported that serum FGF23 levels were related to inflammation and disease activity of RA, and matrix metalloprotease-3 (MMP-3), which is a bone destruction marker, and to bone absorption marker (serum type I collagen cross-linked N-telopeptide [NTx]) but not to bone mineral density (BMD)^2^. As the mechanism linking inflammation and FGF23 has been studied with regard to iron metabolism^3,4^, IL-1^5^β, and IL-6^6^, here we focused on hepcidin as a factor associated with RA inflammation, iron metabolism and systemic osteoporosis.

Hepcidin is a major regulator of iron metabolism and homeostasis and is related to anemia of inflammation^7,8^. Hepcidin is synthesized in hepatocytes and secreted into the bloodstream, where it binds to the iron exporter ferroportin (FPN) in target cells, macrophages, and enterocytes and to some extent in hepatocytes. Hepcidin expression is modulated by iron, inflammation (IL-6), and erythropoiesis. Several recent reports have indicated that iron metabolism can affect bone metabolism^9,10^. Hemochromatosis and thalassemia cause iron overload and osteoporosis^11,12^. Iron overload is a risk factor for progressive bone loss in healthy postmenopausal women and middle-aged men^13^. Furthermore, in one study, lower serum hepcidin levels and higher serum iron levels were reported in patients with osteoporosis than healthy controls^14^.

This study investigated whether iron metabolism and serum hepcidin are related to systemic osteoporosis in patients with RA.

## Results

### Characteristics of the study subjects

The characteristics of the study subjects are listed in Table 1. The mean age was 67.5 ± 11.4 years; 77.5% of the subjects were female and 17.6% were obese (BMI ≧ 25 kg/m^2^). The mean disease duration was 13.6 ± 10.6 years and the mean disease activity scores in 28 joints (DAS28) based on serum CRP and erythrocyte sedimentation rate (ESR) were 2.2 ± 1.5 and 2.6 ± 1.0, respectively. Most patients were classified as being in remission or showing low disease activity, based on the DAS28-CRP and clinical disease activity index (CDAI) (77.1% and 82.4%, respectively). Biological disease-modifying antirheumatic drugs (bDMARDs) including anti-TNF and tocilizumab (TCZ) treatment, were used by 33.6% of subjects, and TCZ was used by 8% of subjects. Active form of vitamin D preparation was prescribed in 14.1% of subjects. The mean eGFR was 73.3 ± 19.6 mL/min/1.73 m^2^, and 26% of the subjects showed an eGFR <60 mL/min/1.73 m^2^. Bisphosphonate or denosumab was used by 63% of subjects. Based on the T score of the femoral neck, osteoporosis and osteopenia were diagnosed in 18.7% and 57.3% of cases, respectively. Based on the T score of the lumbar spine, the respective rates were 22.1% and 36.6%. The T score was normal in the femur in 23.3% of cases, and in the lumbar spine in 41.2% of cases; this difference may have been due to the differential effects of osteoporosis treatment on trabecular and cortical bone. Another possible factor is the progression of vertebral compression and deformity. The mean hemoglobin (Hb) level was 12.5 ± 1.4 g/dL and only a patient showed Hb <9.0 g/dL (which was 8.9 g/dL). No patient was diagnosed with polycythemia. Only 3.4% of our patients used iron agent to treat iron deficiency anemia after being diagnosed by the attending physician.

**Table 1:** Characteristics of the study subjects.

	Mean	±	S.D.	Min	—	Max
Age, years	67.5	±	11.4	22	—	92
Female, *n* (%)	203		(77.5)			
Body mass index, kg/m^2^	21.9	±	3.4	14.4	—	33.5
Body mass index ≥ 25 kg/m^2^, *n* (%)	46		(17.6)			
Disease duration, years	13.6	±	10.6	0	—	54
Rheumatoid factor, U/mL	121.0	±	256.4	0	—	2175
ESR, mm/h	19.4	±	17.8	0	—	110
CRP, mg/dL	0.4	±	0.8	0.0	—	6.3
Matrix metalloprotease 3, mg/mL	122.4	±	100.0	20.0	—	836.7
DAS28-ESR	2.6	±	1.0	0.5	—	6.5
DAS28-CRP	2.2	±	1.5	1.0	—	20.9
SDAI	6.3	±	6.1	0.0	—	37.7
CDAI	5.9	±	5.9	0.0	—	37.4
Disease activity (DAS28-CRP), *n* (%)	high (>4.1), 10 (3.8); moderate (2.7-4.1), 50 (19.1); low (2.3≤, <2.7), 38 (14.5); remission (<2.3), 164 (62.6)					
Disease activity (CDAI), *n* (%)	high (>22), 6 (2.3); moderate (10<, ≤22), 40 (15.3); low (2.8<, ≤10), 123 (46.9); remission (≤2.8), 93 (35.5)					
HAQ-DI	0.5	±	0.7	0	—	3
Prednisolone use, *n* (%)	195		(74.4)			
Daily prednisolone dose, mg/day	3.0	±	3.5	0	—	25
Methotrexate use, *n* (%)	138		(52.7)			
Weekly methotrexate dose, mg/week	4.0	±	4.3	0	—	14
Biological DMARD use, *n* (%)	88		(33.6)			
Infliximab, *n* (%)	24		(9.2)			
Tocilizumab, *n* (%)	21		(8.0)			
Abatacept, *n* (%)	12		(4.6)			
Etanercept, *n* (%)	12		(4.6)			
Golimumab, *n* (%)	8		(3.1)			
Adalimumab, *n* (%)	10		(3.8)			
Certolizumab pegol, *n* (%)	1		(0.4)			
Serum adjusted calcium, mg/dL	9.6	±	0.8	8.6	—	19.6
Serum adjusted calcium level, *n* (%)	high (>10.1), 17 (6.5); normal (8.8-10.1), 239 (91.2); low (<8.8), 6 (2.3)					
Serum phosphate, mg/dL	3.3	±	0.6	1.7	—	4.8
Serum creatinine, mg/dL	0.7	±	0.2	0.4	—	1.7
eGFR, mL/min/1.73 m^2^	73.3	±	19.6	22.9	—	132.3
Red blood cell count, 10^4^/mL	413.0	±	49.4	273	—	571
Hemoglobin, g/dL	12.5	±	1.4	8.9	—	17.6
High level of hemoglobin (>16.8 for male and >14.8 for female), *n* (%)	5		(0.8)			
Low level of hemoglobin (<13.7 for male and <11.6 for female) *n* (%)	92		(35.1)			
Hematocrit, %	37.5	±	3.9	27.3	—	51.3
Platelet count, ×10^4^/mL	22.1	±	6.0	6.6	—	45.2
Low platelet count (<15.8 ×10^4^/mL), *n* (%)	30		(11.5)			
Low platelet count (<10 × 10^4^/mL), *n* (%)	2		(0.8)			
Serum ferritin, ng/mL	62.5	±	63.6	0	—	392
Serum iron, mg/dL	73.0	±	34.5	9	—	200
UIBC, mg/dL	245.6	±	68.5	51	—	458
Femoral T score	−1.5	±	1.0	−4.8	—	1.5
Femoral Z score	0.3	±	1.0	−2.5	—	2.9
Classification of femoral T score, *n* (%)	Osteoporosis, 49 (18.7); osteopenia, 150 (57.3), normal 61 (23.3)					
Lumbar T score	−1.2	±	1.5	−4.8	—	3.8
Lumbar Z score	0.5	±	1.4	−2.7	—	5.2
Classification of lumbar T score, *n* (%)	Osteoporosis, 58 (22.1); osteopenia 96 (36.6); normal 108 (41.2)					
Bone alkaline phosphatase, mg/L	14.4	±	18.2	3.5	—	242.0
TRACP-5b, mU/dL	319.2	±	198.5	18.5	—	1350.0
25(OH)D, ng/mL	16.5	±	6.9	3.5	—	50.3
Hepcidin, ng/mL	14.3	±	19.7	0.0	—	102.3
FGF 23, pg/mL	59.3	±	32.9	0	—	376
Bisphosphonate or denosumab use, *n* (%)	165		(63.0)			
Denosumab use, *n* (%)	8		(3.1)			
Teriparatide use, *n* (%)	9		(3.4)			
Calcium preparation use, *n* (%)	11		(4.2)			
Active form of vitamin D preparation use, *n* (%)	37		(14.1)			
Vitamin K2 use, *n* (%)	8		(3.1)			
Iron agent use, *n* (%)	9		(3.4)			
Annual change of femoral T score*	−0.009	±	0.160			
Annual change of lumbar T score*	0.102	±	0.203			

DAS28, disease activity scores in 28 joints; ESR, erythrocyte sedimentation rate; CRP, C-reactive protein; SDAI, simplified disease activity index; CDAI, clinical disease activity index; HAQ-DI, health assessment questionnaire without disability index; DMARDs, disease-modifying antirheumatic drugs; eGFR, estimated glomerular filtration rate; UIBC, unsaturated iron binding capacity; TRACP-5b, tartrate-resistant acid phosphatase-5b; 25(OH)D, 25-hydroxy vitamin D; FGF23, fibroblast growth factor 23.

*Annual change of T score was evaluated in 231 patients and the mean observational period was 2.0 ± 0.33 years (0.63–3.25).

### Serum hepcidin, serum FGF23, and serum 25(OH)D levels

**Figure 1:**
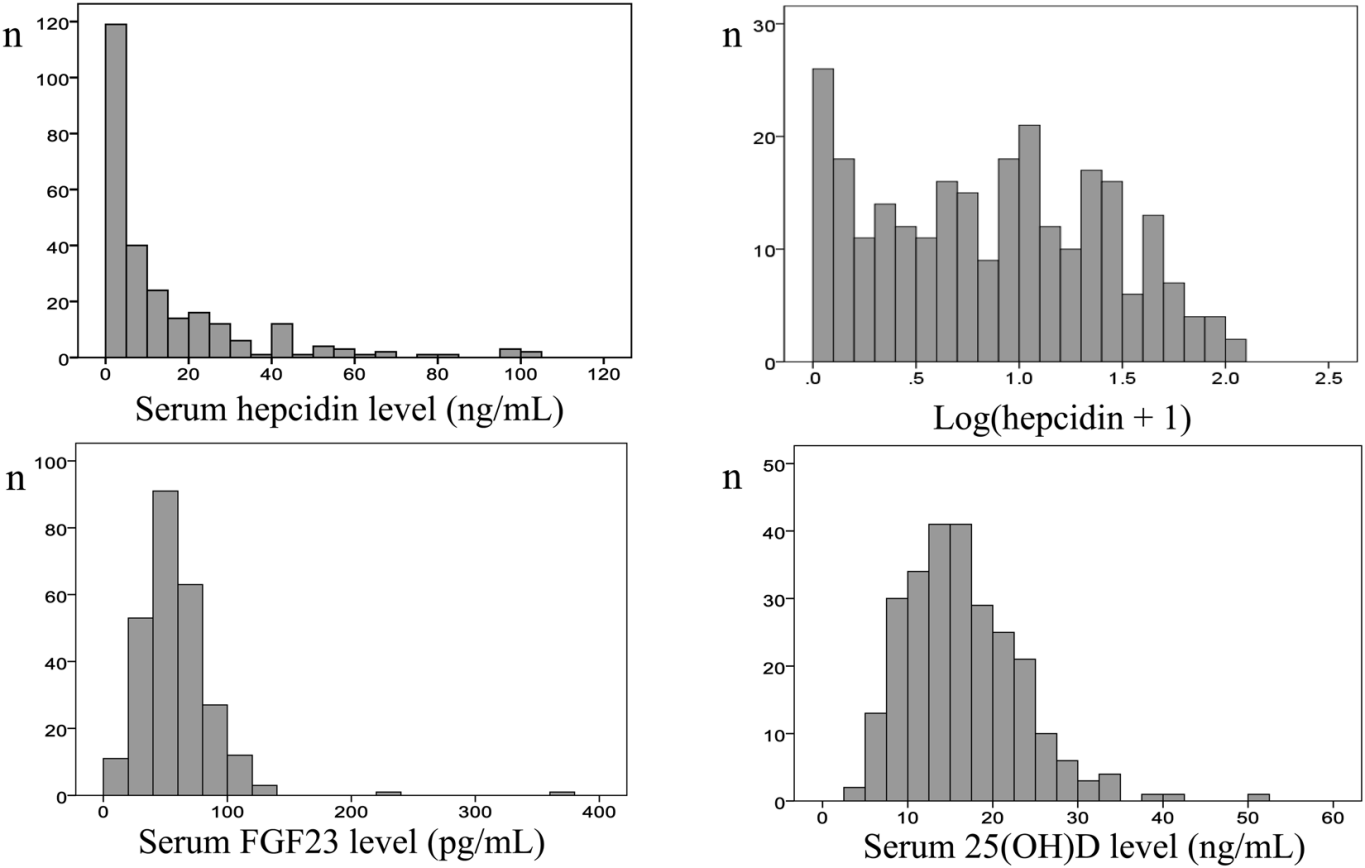
Distributions of serum hepcidin, FGF23, and 25(OH)D levels. As serum hepcidin levels were not normally distributed, the actual hepcidin level + 1 was logarithmically transformed.

The mean serum hepcidin concentration was 14.3 ± 19.7 ng/mL (range, 0.0–102.3 ng/mL) (Table 1). As serum hepcidin levels were not normally distributed, the actual hepcidin level + 1 was logarithmically transformed (Fig. 1). Serum FGF23 and 25-hydroxy vitamin D (25[OH]D) levels were normally distributed (Fig. 1). The serum 25(OH)D levels of most patients (252, 96.2%) were <30 ng/mL; 62 patients (23.7%) had levels 20–30 ng/mL (insufficient), and 190 patients (72.5%) had levels <20 ng/mL (deficient).

### Associations among the serum hepcidin level, iron metabolism, and inflammation

Serum iron, ferritin, and hepcidin levels were significantly positively correlated (Table 2). The Hb level was slightly positively related to these markers of iron metabolism, and platelet count was negatively related to these markers. Markers of inflammation due to RA (DAS28-CRP and serum CRP) were negatively associated with the serum iron level and positively related to serum ferritin and hepcidin levels. With regard to categorical markers, the serum iron level was significantly lower in patients who used PSL than those who did not (83.1 ± 31.4 vs. 69.5 ± 34.9 mg/dL, p = 0.001) (Table 3). Lower serum iron and higher serum ferritin and hepcidin levels were seen in patients who used bDMARDs including TCZ than those who did not (iron, 69.8 ± 32.8 vs. 79.4 ± 36.9 mg/dL, p = 0.046; ferritin, 68.7 ± 69.2 vs. 50.1 ± 48.9 ng/mL, p = 0.023; hepcidin, 15.8 ± 20.2 vs. 11.3 ± 18.4 ng/mL, p = 0.008), and only higher serum iron level was seen in patients who used TCZ (71.2 ± 33.8 vs. 93.4 ± 36.9 mg/dL, p = 0.007).

**Table 2:** Correlations between serum iron, ferritin, and hepcidin levels and other parameters.

	Correlation coefficients			Adjusted correlation coefficients*		
	Fe	Ferritin	Log(hep + 1)	Fe	Ferritin	Log(hep + 1)
Serum iron	1.000	**0.318**	**0.307**	1.000	**0.251**	**0.401**
		(<0.001)	(<0.001)		(<0.001)	(<0.001)
Serum ferritin	**0.318**	1.000	**0.816**	**0.251**	1.000	**0.657**
	(<0.001)		(<0.001)	(<0.001)		(<0.001)
Log(hep + 1)	**0.307**	**0.816**	1.000	**0.401**	**0.657**	1.000
	(<0.001)	(<0.001)		(<0.001)	(<0.001)	
Age	−**0.161**	0.092	0.072	—	—	—
	(0.009)	(0.137)	(0.249)			
Body mass index	−0.021	−0.011	−0.024	—	—	—
	(0.732)	(0.856)	(0.697)			
Disease duration	−0.050	−0.042	−0.023	0.044	0.047	0.057
	(0.416)	(0.494)	(0.707)	(0.489)	(0.460)	(0.370)
eGFR	0.055	0.002	0.004	—	—	—
	(0.378)	(0.980)	(0.946)			
Red blood cell	0.024	−0.121	−**0.123**	−0.006	−**0.139**	−0.101
	(0.694)	(0.050)	(0.047)	(0.922)	(0.028)	(0.110)
Hemoglobin	**0.400**	**0.196**	**0.125**	**0.420**	**0.127**	**0.243**
	(<0.001)	(0.001)	(0.043)	(<0.001)	(0.045)	(<0.001)
Hematocrit	**0.305**	0.120	0.054	**0.318**	0.060	**0.138**
	(<0.001)	(0.052)	(0.383)	(<0.001)	(0.347)	(0.029)
Platelet	−**0.241**	−**0.173**	−**0.172**	−**0.186**	−0.064	−**0.203**
	(<0.001)	(0.005)	(0.005)	(0.003)	(0.315)	(0.001)
UIBC	−**0.517**	−**0.707**	−**0.708**	−**0.646**	−**0.499**	−**0.669**
	(<0.001)	(<0.001)	(<0.001)	(<0.001)	(<0.001)	(<0.001)
DAS28-ESR	−**0.316**	0.109	0.100	−**0.236**	**0.184**	0.030
	(<0.001)	(0.079)	(0.108)	(<0.001)	(0.003)	(0.633)
DAS28-CRP	−**0.223**	**0.151**	**0.133**	−0.100	**0.140**	0.091
	(<0.001)	(0.014)	(0.032)	(0.114)	(0.026)	(0.152)
SDAI	−**0.175**	0.107	0.093	−0.100	**0.176**	0.034
	(0.004)	(0.083)	(0.132)	(0.114)	(0.005)	(0.588)
CDAI	−**0.148**	0.094	0.068	−0.095	**0.172**	0.034
	(0.016)	(0.131)	(0.273)	(0.133)	(0.06)	(0.596)
HAQ-DI	−**0.167**	−0.068	−0.047	−**0.138**	0.005	−0.117
	(0.007)	(0.281)	(0.451)	(0.031)	(0.933)	(0.068)
Rheumatoid factor	0.014	0.061	0.029	0.003	0.028	0.005
	(0.823)	(0.324)	(0.636)	(0.960)	(0.665)	(0.940)
ESR	−**0.344**	0.112	**0.143**	−**0.178**	**0.178**	0.075
	(<0.001)	(0.070)	(0.021)	(0.005)	(0.005)	(0.241)
CRP	−**0.235**	**0.219**	**0.257**	—	—	—
	(<0.001)	(<0.001)	(<0.001)			
MMP-3	−**0.206**	0.051	−0.005	−0.053	**0.187**	−0.010
	(0.001)	(0.412)	(0.930)	(0.402)	(0.003)	(0.874)
Daily PSL dose	−**0.197**	−0.049	−**0.163**	−0.320	0.052	−0.115
	(0.001)	(0.433)	(0.008)	(0.617)	(0.413)	(0.058)
Weekly MTX dose	0.004	0.028	−0.056	−0.043	0.120	−0.0r06
	(0.954)	(0.655)	(0.371)	(0.505)	(0.058)	(0.930)
Serum bone Alkaline phosphatase	−0.055	−0.003	−0.001	0.098	−0.024	−0.034
	(0.376)	(0.957)	(0.986)	(0.122)	(0.710)	(0.588)
Serum TRACP-5b	−**0.130**	−0.008	−0.018	−0.0749	−0.074	−0.053
	(0.035)	(0.894)	(0.777)	(0.227)	(0.244)	(0.405)
Serum adjusted calcium	0.013	0.086	0.071	−0.007	0.094	0.056
	(0.830)	(0.165)	(0.249)	(0.910)	(0.139)	(0.378)
Serum phosphate	0.000	−0.021	0.063	−0.043	0.011	0.083
	(0.998)	(0.730)	(0.310)	(0.497)	(0.861)	(0.193)
Serum 25(OH)D	0.046	**0.250**	**0.193**	0.042	**0.200**	**0.202**
	(0.461)	(<0.001)	(0.002)	(0.510)	(0.001)	(0.001)
Serum FGF 23	0.009	0.040	−0.019	0.089	0.108	0.029
	(0.889)	(0.523)	(0.763)	(0.160)	(0.089)	(0.644)
Femoral T score	**0.230**	0.006	−0.038	**0.127**	0.022	0.002
	(<0.001)	(0.919)	(0.543)	(0.045)	(0.726)	(0.973)
Femoral Z score	**0.150**	0.067	0.044	**0.126**	0.041	0.044
	(0.015)	(0.279)	(0.479)	(0.048)	(0.516)	(0.487)
Lumbar T score	0.067	−0.044	−0.110	−0.047	0.028	−0.072
	(0.283)	(0.480)	(0.076)	(0.459)	(0.658)	(0.3257)
Lumbar Z score	0.004	−0.002	−0.051	−0.055	0.039	−0.043
	(0.953)	(0.973)	(0.410)	(0.389)	(0.188)	(0.501)
Annual change of femoral T score	0.071	−0.104	−0.031	0.001	−0.132	−0.021
	0.284	0.115	0.640	(0.989)	(0.050)	(0.762)
Annual change of lumbar T score	0.046	**0.135**	**0.146**	0.011	0.086	**0.151**
	0.486	0.041	0.026	(0.869)	(0.206)	(0.026)

*Adjusted for age, sex, BMI, eGFR, CRP, PSL use, bDMARD use, anti-resorption drug use, teriparatide use, and iron agent use.

eGFR, estimated glomerular filtration rate; UIBC, unsaturated iron binding capacity; DAS28, disease activity scores in 28 joints; ESR, erythrocyte sedimentation rate; CRP, C-reactive protein; SDAI, simplified disease activity index; CDAI, clinical disease activity index; HAQ-DI, health assessment questionnaire without disability index; MMP-3, matrix metalloprotease 3; PSL, prednisolone; MTX, methotrexate; TRACP-5b, tartrate-resistant acid phosphatase-5b; 25(OH)D, 25-hydroxy vitamin D; FGF23, fibroblast growth factor 23.

Annual change of T score was evaluated in 231 patients and the mean observational period was 2.0 ± 0.33 years (0.63–3.25).

**Table 3:** Relationships between serum iron, ferritin, and hepcidin and categorical variables.

	**Female**		**Male**		
Iron, mg/dL	203	72.1 ± 33.2	59	76.0 ± 38.8	0.643
Ferritin, ng/mL	203	59.4 ± 60.9	59	73.2 ± 71.6	0.073
Hepcidin, ng/mL	203	13.9 ± 18.9	59	15.4 ± 22.3	0.905
	**Anti-resorption treatment (−)**		**Anti-resorption treatment (+)**		
Iron, mg/dL	97	71.8 ± 34.6	165	73.7 ± 34.5	0.650
Ferritin, ng/mL	97	70.6 ± 73.5	165	57.7 ± 56.7	0.142
Hepcidin, ng/mL	97	16.1 ± 21.5	165	13.2 ± 18.6	0.193
	**Vitamin D treatment (−)**		**Vitamin D treatment (+)**		
Iron, mg/dL	225	72.8 ± 34.4	37	74.1 ± 35.5	0.961
Ferritin, ng/mL	225	60.0 ± 62.6	37	77.8 ± 68.4	0.050
Hepcidin, ng/mL	225	13.8 ± 19.4	37	17.2 ± 21.4	0.334
	**PSL (−)**		**PSL (+)**		
**Iron, mg/dL**	**67**	**83.1** ± **31.4**	**195**	**69.5** ± **34.9**	**0.001**
Ferritin, ng/mL	67	63.6 ± 54.6	195	62.1 ± 66.5	0.222
Hepcidin, ng/mL	67	13.0 ± 12.3	195	14.7 ± 21.7	0.083
	**MTX (−)**		**MTX (+)**		
Iron, mg/dL	124	72.8 ± 35.4	138	73.2 ± 33.8	0.930
Ferritin, ng/mL	124	55.8 ± 52.9	138	68.5 ± 71.5	0.332
Hepcidin, ng/mL	124	13.5 ± 19.0	138	15.0 ± 20.4	0.938
	**bDMARDs (−)**		**bDMARDs (+)**		
**Iron, mg/dL**	**174**	**69.8** ± **32.8**	**88**	**79.4** ± **36.9**	**0.046**
**Ferritin, ng/mL**	**174**	**68.7** ± **69.2**	**88**	**50.1** ± **48.9**	**0.023**
**Hepcidin, ng/mL**	**174**	**15.8** ± **20.2**	**88**	**11.3** ± **18.4**	**0.008**
	**Tocilizumab (−)**		**Tocilizumab (+)**		
**Iron, mg/dL**	**241**	**71.2** ± **33.8**	**21**	**93.4** ± **36.9**	**0.007**
Ferritin, ng/mL	241	64.4 ± 65.6	21	40.4 ± 24.2	0.330
Hepcidin, ng/mL	241	14.9 ± 20.2	21	7.5 ± 10.0	0.174
	**Iron agent (−)**		**Iron agent (+)**		
Iron, mg/dL	253	72.7 ± 34.8	9	81.6 ± 25.3	0.238
Ferritin, ng/mL	253	61.2 ± 62.6	9	99.7 ± 83.5	0.142
Hepcidin, ng/mL	253	14.1 ± 19.9	9	19.8 ± 14.0	0.055

Data are numbers of patients, mean ± S.D.

Anti-resorption treatment included bisphosphonate and denosumab use. bDMARDs included anti-TNF and tocilizumab treatment. Vitamin D treatment was the use of active form of vitamin D preparation.

PSL, prednisolone; MTX, methotrexate; DMARDs, disease-modifying antirheumatic drugs.

After stratification according to disease activity, serum iron level was positively associated with serum hepcidin level in patients in remission (CDAI ≦2.8) (r = 0.518, p < 0.001, n = 93) and those showing low disease activity (CDAI 2.8 < , ≤10) (r = 0.242, p = 0.007, n = 123) but not in those showing in moderate to high disease activity (CDAI 10 < ) (r = 0.177, p = 0.239, n = 36) (Table S1).

### Associations among the serum hepcidin level, iron metabolism, and osteoporosis

**Figure 2:**
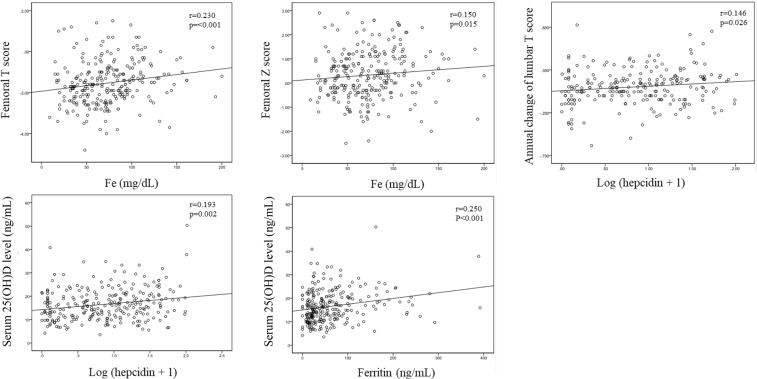
Scatter plots of iron metabolism and osteoporosis markers.

With regard to osteoporosis and markers of bone metabolism, the serum iron level was positively related to femoral T and Z scores (r = 0.230, p < 0.001 and r = 0.150, p = 0.015, respectively), and ferritin and hepcidin levels were positively related to the 25(OH)D level (r = 0.250, p < 0.001 and r = 0.193, p = 0.002, respectively) (Table 2 and Fig. 2). Annual change of lumbar T score, but not that of femoral T score, was positively related to hepcidin level (r = 0.146, p = 0.026) (Table 2 and Fig. 2). After adjustment for age, sex, BMI, eGFR, CRP, the use of bDMARD, PSL, anti-bone resorption drug, teriparatide, and iron agent, those significant relationships were still remained (Table 2). Serum bone alkaline phosphatase, tartrate-resistant acid phosphatase-5b (TRACP-5b) and FGF23 levels were not related to markers of iron metabolism (Table 2).

Multiple regression analyses adjusted for age, sex, BMI, eGFR, CRP, the use of bDMARD, PSL, anti-bone resorption drug, teriparatide, and iron agent, indicated that the serum iron level was positively related to femoral T and Z scores (β = 0.121, p = 0.039 and β = 0.123, p = 0.046, respectively), and serum ferritin and hepcidin levels were positively related to the serum 25(OH)D level (β = 0.200, p = 0.002 and β = 0.207, p = 0.002, respectively). Annual change of lumbar T score, but not that of femoral T score, was positively related to serum hepcidin level (β = 0.159, p = 0.025). MMP-3 was also positively related to the serum ferritin level (β = 0.160, p = 0.004), whereas FGF23, bone alkaline phosphatase and TRACP-5b were not related to any of these three variables (Table 4).

**Table 4:** Relationships between serum iron, ferritin, and hepcidin levels and osteoporosis-related factors analyzed by multiple regression.

	Fe		Ferritin		Log (hep + 1)	
	β	*p*	β	*p*	β	*p*
Femoral T score	**0.121**	**0.039**	0.028	0.631	0.012	0.840
Femoral Z score	**0.123**	**0.046**	0.048	0.449	0.059	0.362
Bone alkaline phosphatase	0.101	0.118	−0.027	0.677	−0.040	0.543
TRACP-5b	−0.067	0.294	−0.079	0.222	−0.058	0.383
25(OH)D	0.044	0.493	**0.200**	**0.002**	**0.207**	**0.002**
FGF-23	0.090	0.146	0.103	0.096	0.029	0.653
MMP-3	−0.046	0.402	**0.160**	**0.004**	−0.015	0.790
Annual change of femoral T score*	0.000	0.996	−0.132	0.056	−0.031	0.659
Annual change of lumbar T score*	0.013	0.851	0.085	0.225	**0.159**	**0.025**

Multiple regression models were adjusted for age, sex, BMI, eGFR, CRP, PSL use, bDMARD use, anti-resorption drug use, teriparatide use, and iron agent use.

TRACP-5b, tartrate-resistant acid phosphatase-5b; 25(OH)D, 25-hydroxy vitamin D; FGF23, fibroblast growth factor 23; MMP-3, matrix metalloprotease 3.

*Annual change of T score was evaluated in 231 patients and the mean observational period was 2.0 ± 0.33 years (0.63–3.25).

After stratification according to disease activity, a positive relationship between serum iron and the femoral T score was observed (r = 0.318, p = 0.002), and between serum hepcidin and the annual change in lumbar T score (r = 0.293, p = 0.010) in patients in remission (CDAI ≦2.8, n = 93) (Table S2). Serum 25(OH)D level was positively associated with serum ferritin and serum hepcidin levels in patients in remission (r = 0.356, p < 0.001 and r = 0.342, p = 0.001; respectively, n = 93) and in those showing low disease activity (r = 0.293, p = 0.001 and r = 0.204, p = 0.023, respectively; n = 123) but not in those showing moderate to high disease activity (Table S2).

### Associations between serum hepcidin level and other parameters

**Figure 3:**
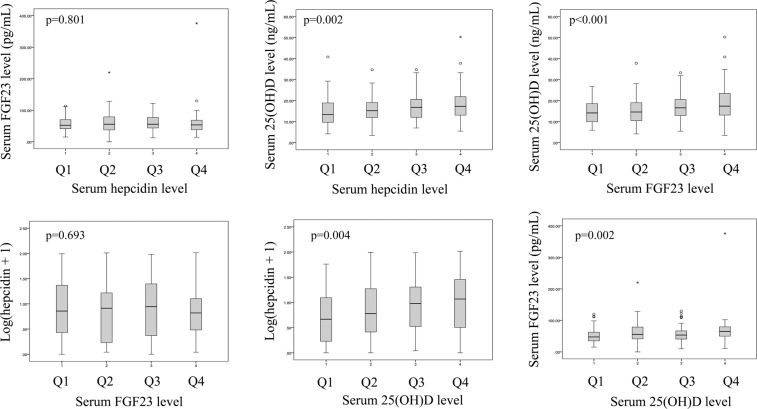
Relationships among serum hepcidin, FGF23, and 25(OH)D levels. Each factor was divided into four groups (Q1–Q4), and distributions of other factors are indicated in relation to each quartile.

All subjects were divided into four groups according to serum hepcidin level (Q1, –1.3 ng/mL; Q2, 1.4–6.6 ng/mL; Q3, 6.7–19.5 ng/mL; Q4, 20.0–102.3 ng/mL; Table S3). Higher hepcidin levels were significantly related to greater inflammation, a higher ferritin level, a higher iron level, lower unsaturated iron binding capacity (UIBC), higher 25(OH)D level, and annual change of lumbar T score (Table S3, Fig. 3). Lower hepcidin levels were significantly related to a higher daily PSL dose, a higher rate of bDMARD use, a higher platelet count, and a lower Hb level. Age, disease duration, renal function, TCZ use, BMD, markers of bone metabolism, and FGF23 level were not related to the serum hepcidin level (Table S3, Fig. 3). Iron metabolism, inflammation due to RA, and the 25(OH)D level were significantly related to the serum hepcidin level.

### Associations between the serum 25(OH)D level and other parameters

All subjects were divided into four groups according to serum 25(OH)D level (Q1, 3.5–11.6 ng/mL; Q2, 11.7–15.6 ng/mL; Q3, 15.8–20.6 ng/mL; Q4, 20.7–50.3 ng/mL; Table S4). Higher 25(OH)D levels were significantly related to older age, a higher adjusted Ca level, a lower eGFR level, a higher ferritin level, a higher hepcidin level, a higher Z score of femoral neck, a higher FGF23 level and a higher annual change of lumbar T score (Table S4, Fig. 3). Inflammation, disease activity, and treatments were not associated with serum 25(OH)D level. The same analyses were performed on patients who did not use active vitamin D (non-VD users) only, and similar results were obtained (Table S5).

### Associations between the serum FGF23 level and other parameters

Subjects were divided into quartile groups according to the serum FGF23 level (Q1, 0–40.3 pg/mL; Q2, 40.7–54.0 pg/mL; Q3, 54.1–73.1 pg/mL; Q4, 73.2–376 pg/mL; Table S6). Higher FGF23 levels were significantly associated with older age, a higher BMI, higher DAS28-ESR, MMP-3, and serum-adjusted Ca levels, lower eGFR and Hb levels, and a higher 25(OH)D level (Table S6, Fig. 3). Serum hepcidin level was not associated with FGF23 level.

## Discussion

The results of this study indicate that the serum iron level is positively related to BMD and serum hepcidin and ferritin levels are positively related to the 25(OH)D level, which is positively related to the femoral Z score. As to 231 patients re-examined BMD, annual change of lumbar T score was positively related to serum hepcidine level and higher 25(OH)D quartiles. The serum hepcidin level and iron metabolism indirectly and directly affected osteoporosis in patients with RA.

Researchers have reported serum hepcidin concentrations in patients with RA, focusing on anemia. In studies, the serum hepcidin level was higher in patients with RA compared to healthy subjects^15,16^, which may be the result of inflammation due to RA. Iron deficiency, which often accompanies RA, is also an essential factor influencing serum hepcidin. In one study, patients with RA and iron deficiency had significantly decreased serum hepcidin levels compared to those with RA and anemia with chronic inflammation^17^. As hepcidin is influenced by inflammation and iron metabolism, results differ on the influence on Hb and disease activity associated with hepcidin on RA^16,18^. Furthermore, in the same study of patients with RA, iron metabolism was related to serum hepcidin levels cross-sectionally, but inflammation was related longitudinally^19^. Anti-IL-6 therapy decreases serum hepcidin and improves the Hb level^20–22^, and the effect is more marked with anti-IL-6 than anti-TNF therapy^20^. In our cross-sectional study, CRP and Hb were related to the serum hepcidin level, but stronger relationships were observed for iron metabolism (i.e., ferritin, iron, and UIBC). Moreover, positive relationships were found among serum hepcidin, ferritin, iron and Hb levels which seemed like iron deficiency anemia pattern. The reason for less influence from inflammation was considered because most patients in this study mainly showed low disease activity or were in remission. Also a high percentage of patients in the lower quartile of serum hepcidin level were treated with bDMARDs. Thus, the serum hepcidin level seemed to be closely reflected by iron metabolism, including in patients showing low disease activity or in remission.

Iron accumulation is a risk factor for osteoporosis, and hepcidin is expected to be a useful therapeutic target^23–25^. In one study, hepcidin knockout mice had a higher serum ferritin level and higher iron in the liver and femur than controls and showed low bone mass and changes in bone microarchitecture^26^. Hepcidin knockout mice also showed a marked reduction in bone load-bearing capacity with enhanced bone resorption^23^. A mouse model with overexpression of hepcidin showed higher levels of serum hepcidin and lower levels of serum ferritin, and bone loss and changes in markers of bone metabolism after ovariectomy were ameliorated^24^. In humans, genetic hemochromatosis and thalassemia cause iron overload, and osteoporosis is a major complication^11,12^. Iron overload (an elevated ferritin level) is a risk factor for progressive bone loss in healthy postmenopausal women and middle-aged men and a risk factor for radiological vertebral fracture in postmenopausal women^13^. Liu *et al*. compared serum hepcidin levels in 40 patients with osteoporosis and 40 healthy controls^14^. They identified lower serum hepcidin levels and higher iron levels in patients with osteoporosis compared to healthy controls, and the serum hepcidin level was negatively related to the serum iron level^14^. In this study, the serum hepcidin level was positively related to serum iron and ferritin levels, and serum iron levels were positively related to BMD, in contrast to the studies outlined above. Moreover, annual change of lumbar T score, not femoral, was positively related to serum hepcidin and 25(OH)D levels. No direct relations were found between markers of bone metabolism and the serum hepcidin level. Those findings were more apparent in patients who were in remission. The unexpected results about iron metabolism and osteoporosis in this study seemed because the relationships between serum iron and hepcidin levels and inflammation due to RA, bDMARD treatment, and iron deficiency in patients with RA are complicated. The disease activity of most patients in this study was low; the results may have been different in patients with higher RA activity.

Vitamin D deficiency is a risk factor for autoimmune disorders, including RA^27^. Vitamin D affects bone mineralization and calcium regulation, and the serum level of 25(OH)D in RA is positively related to BMD^28^. A recent *in vitro* study suggested that binding of 1,25(OH)_2_D to the vitamin D receptor directly suppressed hepcidin gene transcription^29^. Furthermore, supplementation of vitamin D reduces serum hepcidin levels in healthy subjects^29,30^, patients with chronic kidney disease^29,31^, and pediatric patients with inflammatory bowel disease^32^. In this study, the serum 25(OH)D level was not related to disease activity but was negatively related to renal function and positively related to the serum adjusted Ca level, serum ferritin level, serum hepcidin level, serum FGF23 level, and femoral Z score. There are some possible explanations for the positive association between 25(OH)D and hepcidin. First, the results of cross-sectional studies of the relationship between hepcidin and 25(OH)D are inconsistent. One study of children with inflammatory bowel disease suggested that a higher 25(OH)D concentration was related to a lower hepcidin level^33^. Another study of older Mexican adults indicated that the serum hepcidin level did not differ between patients with 25(OH)D ≥ 50 nmol/L and <50 nmol/L^34^. The characteristics of subjects were markedly different in these two studies and the present study. Second, race may influence the association between vitamin D deficiency and anemia^35^. In one study, serum 25(OH)D < 50 nmol/L was significantly associated with anemia among black but not white subjects^35^. Further research is needed to reach definitive conclusions.

In this study, a higher FGF23 level was related to less kidney function and older age but not to serum hepcidin level. As we reported previously^2^, RA disease activity and MMP-3 are positively related to serum FGF23 levels. Meanwhile, this study showed that higher FGF23 levels are related to lower Hb levels and to higher 25(OH)D and adjusted Ca levels. This study and the previous study differed with regard to the subjects; the previous study included patients with higher disease activity (CRP, 3.2 ± 3.4 mg/dL; DAS28-ESR, 4.7 ± 1.4; bDMARD use, 6.6%) than patients in the present study.

**Figure 4:**
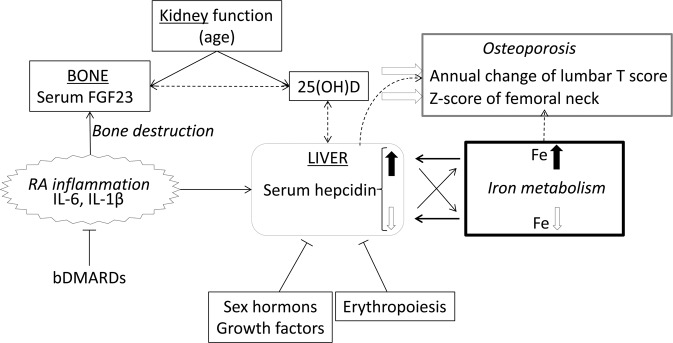
Estimated relationships related to hepcidin in patients with RA. Hepcidin is usually regulated by iron metabolism—iron overload (black arrow) leads to an increase in hepcidin, and iron deficiency (white arrow) leads to a decrease—and is suppressed by erythropoiesis, sex hormones, and growth factors. To maintain iron homeostasis, higher hepcidin levels result in a decrease in serum iron levels, while lower hepcidin levels increase serum iron levels. When inflammation due to RA occurs, production of hepcidin is increased through inflammatory cytokines that cause elevated ferritin levels. In this study, serum iron levels are positively related to serum hepcidin and ferritin levels but negatively related to inflammation due to RA. This unexpected relationship may give arisen because inflammation in most of the patients was well-controlled. Although iron overload and hepcidin may influence osteoporosis, in this study serum iron level was positively related to BMD but serum hepcidin level was not. However, serum 25(OH)D level was positively related to the serum hepcidin level and also positively related to the femoral Z score in this study. According to previous reports, opposite effects of serum iron level on BMD and 25(OH)D on hepcidin are indicated, and further research is needed to determine the mechanism. Serum FGF23 is not directly related to the serum hepcidin level, but serum 25(OH)D level and inflammation are common factors involved in the regulation of both serum hepcidin and FGF23 levels.

Finally, we investigated relationships among serum hepcidin, iron metabolism and osteoporosis in patients with RA (Fig. 4). Hepcidin is usually regulated by iron metabolism (iron overload leads to an increase in hepcidin, and iron deficiency leads to a decrease) and is suppressed by erythropoiesis, sex hormones, and growth factors^36^. Hepcidin deficiency due to genetic hemochromatosis or severe liver dysfunction leads to higher iron levels, while higher hepcidin levels decrease iron levels, as seen in chronic inflammation and some cancers. When inflammation occurs due to RA, the production of hepcidin increases due to the expression of inflammatory cytokines, and affects to decreasing iron and increasing ferritin levels. In this study of RA patients in remission or showing low disease activity, the serum iron level was positively associated with serum hepcidin and ferritin levels, but was negatively associated with inflammation due to RA. Although iron overload and hepcidin may influence osteoporosis, the serum iron level was positively related to BMD in this study but serum hepcidin and ferritin levels were not. However, the serum 25(OH)D level was positively related to the serum hepcidin level and also positively related to femoral Z scores. Serum hepcidin level was also positively related to the annual change of lumbar T score. In contrast to previous reports, opposite effects of the serum iron level to BMD and the serum 25(OH)D level to the serum hepcidin level were indicated, and further research is needed to determine the mechanisms. However, the serum FGF23 level was not directly related to the serum hepcidin level, but the serum 25(OH)D level and inflammation were common factors related to both serum hepcidin and FGF23 levels.

The major limitation of this study is single-center nature and the absence of healthy controls. Blood samples were not collected during fasting so an effect of diet cannot be ruled out. We could not analyze the patient background of smoking, alcohol intake, menopausal status and the use of hormone replacement therapy. Also, physical activity and body weight bearing activity were not included, but health assessment questionnaire without disability index (HAQ-DI) and BMI could replace them. We investigated only serum markers and BMD but not fracture data. Further studies are needed regarding to fragility fractures controlling patients’ background. The number of patients using iron agents was low and no significant effect was observed.

In conclusion, the serum iron level was positively related to BMD in these patients with RA, and a higher iron level was not considered a risk factor for osteoporosis. The serum hepcidin level was not related to BMD and markers of bone metabolism but was positively related to the serum 25(OH)D level, which was positively related to the femoral Z score. And the serum hepcidin level was positively related to the annual change of lumbar T score. The serum FGF23 level was not associated with the serum hepcidin level. Serum hepcidin and serum iron were indirectly and directly related to osteoporosis in this study of patients with RA.

## Methods

### Subjects

The study population consisted of 262 patients with RA treated at the Niigata Rheumatic Center between December 2015 and October 2016. The inclusion criterion was meeting the 1987 American Rheumatism Association criteria for RA^37^ and/or the 2010 American College of Rheumatology (ACR)/European League Against Rheumatism (EULAR) RA criteria^38^. A total of 263 patients consented to participate in this study and 1 patient was excluded because of an elevated ferritin level (2,654 ng/mL). Blood samples were taken in either the morning or afternoon. RA activity and treatments were reviewed through medical records.

The study protocol was approved by the ethics committee of the Niigata Rheumatic Center and performed in accordance with the Declaration of Helsinki. Written informed consent was obtained from all participants.

### Calculation of estimated glomerular filtration rates and serum adjusted calcium levels

Estimated glomerular filtration rates (eGFRs) were calculated using the standard formula for females, 194 × Cr^−1.094^ × Age^−0.287^ × 0.739, which was developed using the inulin clearance of Japanese subjects as a standard^39^. Serum adjusted calcium levels were calculated if serum albumin levels were below 4 g/dL as follows: actual calcium level + 4 – serum albumin level^40^.

### Biochemical assays of hepcidin, FGF23, and 25(OH)D, and reference ranges of bone metabolic markers

Hepcidin, FGF23, and 25-hydroxy vitamin D (25[OH]D) concentrations were determined using serum samples collected and stored at −80 °C. The serum hepcidin level using liquid chromatography-tandem mass spectrometry (Medical Care Proteomics Biotechnology, Ishikawa, Japan). The serum FGF23 level was measured using sandwich enzyme-linked immunosorbent assay (Kainos Laboratories, Tokyo, Japan), which detected full-length human FGF23^41^. 25(OH)D was quantified by electrochemiluminescence immunoassay (ECLIA; Roche Diagnostics, Tokyo, Japan).

Reference ranges of bone metabolic markers were follows; bone alkaline phosphatase, 3.7–20.9 μg/L for men, 2.9–14.5 μg/L for premenopausal women and 3.8–22.6 μg/L for postmenopausal women; tartrate-resistant acid phosphatase-5b (TRACP-5b), 170–590 mU/dL for men and 120–420 mU/dL for women; matrix metalloprotease 3 (MMP-3), 35.2–123.8 ng/mL for men and 16.1–56.8 ng/mL for women. The serum 25(OH)D levels were classified as follows: 30 ≤ ng/mL, sufficient; 20 ≤ and < 30 ng/mL, insufficient; <20 ng/mL, deficient.

### Bone mineral density measurement

Bone mineral density (BMD) using dual-energy X-ray absorptiometry (PRODIGY; GE Healthcare, Madison, WI, USA). Lumbar spine L1–4 and basically left, but if impossible right femoral neck, were evaluated at the recruitment in all patients. The T score represents the difference from the mean BMD of young healthy subjects (in terms of standard deviation), and the Z score is obtained based on comparison with age- and sex-matched controls. The BMD T scores were classified as follows: ≦−2.5, osteoporosis; −2.5< and < −1, osteopenia; −1≦, normal. Two hundred and thirty-one patients were re-examined BMD in about three years and annual change of T score was evaluated. The mean observational period was 2.0 ± 0.33 years (0.63–3.25).

### Statistical analyses

Most data are expressed as the mean ± standard deviation or number (%). As serum hepcidin levels were not normally distributed, the actual hepcidin level + 1 was logarithmically transformed. Correlation coefficients were obtained using Spearman’s rank method and also correlation coefficients adjusted for age, sex, body mass index (BMI), eGFR, C-reactive protein (CRP), and use of biological disease-modifying antirheumatic drugs (bDMARDs) were calculated. Two groups of categorical variables were compared using the Mann–Whitney U test. Multiple regression analyses were performed with adjustment for the following factors: age, sex, BMI, eGFR, CRP, and the use of bDMARD, PSL, anti-bone resorption drug, teriparatide, and iron agent. Serum hepcidin, FGF23, and 25(OH)D levels were divided into four groups as Q1–Q4 using SPSS software, and the associations between these quartiles and other parameters were analyzed with the Jonckheere-Terpstra trend test.

All statistical analyses were performed with SPSS (ver. 19; IBM, Chicago, IL, USA). In all analyses, *p* < 0.05 was taken to indicate statistical significance.

## Supplementary information

Supplementary information.

## References

[1] Tanaka S, Fujita S, Kizawa S, Morita H, Ishizaka N (2016). Association between FGF23, alpha-Klotho, and Cardiac Abnormalities among Patients with Various Chronic Kidney Disease Stages. PLoS One.

[2] Sato H (2016). Serum Fibroblast Growth Factor 23 (FGF23) in Patients with Rheumatoid Arthritis. Intern Med.

[3] Wolf, M., Koch, T.A. & Bregman, D.B. Effects of iron deficiency anemia and its treatment on fibroblast growth factor 23 and phosphate homeostasis in women. *J Bone Miner Res* (2013). 10.1002/jbmr.1923 23505057

[4] Braithwaite V, Prentice AM, Doherty C, Prentice A (2012). FGF23 is correlated with iron status but not with inflammation and decreases after iron supplementation: a supplementation study. Int J Pediatr Endocrinol.

[5] Yamazaki M (2015). Interleukin-1-induced acute bone resorption facilitates the secretion of fibroblast growth factor 23 into the circulation. J Bone Miner Metab.

[6] Durlacher-Betzer K (2018). Interleukin-6 contributes to the increase in fibroblast growth factor 23 expression in acute and chronic kidney disease. Kidney Int.

[7] Singh B, Arora S, Agrawal P, Gupta SK (2011). Hepcidin: a novel peptide hormone regulating iron metabolism. Clin Chim Acta.

[8] Sebastiani G, Wilkinson N, Pantopoulos K (2016). Pharmacological Targeting of the Hepcidin/Ferroportin Axis. Front Pharmacol.

[9] Chen B, Li GF, Shen Y, Huang XI, Xu YJ (2015). Reducing iron accumulation: A potential approach for the prevention and treatment of postmenopausal osteoporosis. Exp Ther Med.

[10] Smith JT, Schneider AD, Katchko KM, Yun C, Hsu EL (2017). Environmental Factors Impacting Bone-Relevant Chemokines. Front Endocrinol (Lausanne).

[11] Dede AD (2016). Thalassemia-associated osteoporosis: a systematic review on treatment and brief overview of the disease. Osteoporos Int.

[12] Valenti L (2009). Association between iron overload and osteoporosis in patients with hereditary hemochromatosis. Osteoporos Int.

[13] Kim BJ (2012). Iron overload accelerates bone loss in healthy postmenopausal women and middle-aged men: a 3-year retrospective longitudinal study. J Bone Miner Res.

[14] Liu B (2018). Reduced hepcidin level features osteoporosis. Exp Ther Med.

[15] Sitara D (2004). Homozygous ablation of fibroblast growth factor-23 results in hyperphosphatemia and impaired skeletogenesis, and reverses hypophosphatemia in Phex-deficient mice. Matrix Biol.

[16] Ostgard RD (2017). Hepcidin plasma levels are not associated with changes in haemoglobin in early rheumatoid arthritis patients. Scand J Rheumatol.

[17] van Santen S (2011). Hepcidin and hemoglobin content parameters in the diagnosis of iron deficiency in rheumatoid arthritis patients with anemia. Arthritis Rheum.

[18] Sahebari M (2018). Serum hepcidin level and rheumatoid arthritis disease activity. Eur J Rheumatol.

[19] Padjen I (2017). Clinical meaning and implications of serum hemoglobin levels in patients with rheumatoid arthritis. Semin Arthritis Rheum.

[20] Song SN (2013). Comparative evaluation of the effects of treatment with tocilizumab and TNF-alpha inhibitors on serum hepcidin, anemia response and disease activity in rheumatoid arthritis patients. Arthritis Res Ther.

[21] Suzuki S (2017). Hepcidin-25 gives an indication of the therapeutic effectiveness of tocilizumab in rheumatoid arthritis - Relationship between disease activity of rheumatoid arthritis and anemia. Rev Bras Reumatol Engl Ed.

[22] Isaacs JD, Harari O, Kobold U, Lee JS, Bernasconi C (2013). Effect of tocilizumab on haematological markers implicates interleukin-6 signalling in the anaemia of rheumatoid arthritis. Arthritis Res Ther.

[23] Sun L (2014). Hepcidin deficiency undermines bone load-bearing capacity through inducing iron overload. Gene.

[24] Zhang P (2018). Hepcidin is an endogenous protective factor for osteoporosis by reducing iron levels. J Mol Endocrinol.

[25] Li GF, Pan YZ, Sirois P, Li K, Xu YJ (2012). Iron homeostasis in osteoporosis and its clinical implications. Osteoporos Int.

[26] Shimada T (2004). FGF-23 is a potent regulator of vitamin D metabolism and phosphate homeostasis. J Bone Miner Res.

[27] Harrison, S.R., Li, D., Jeffery, L.E., Raza, K. & Hewison, M. Vitamin D, Autoimmune Disease and Rheumatoid Arthritis. *Calcif Tissue Int* (2019). 10.1007/s00223-019-00577-2 PMC6960236 31286174

[28] Chen J (2014). Vitamin D deficiency and low bone mineral density in native Chinese rheumatoid arthritis patients. Int J Rheum Dis.

[29] Bacchetta J (2014). Suppression of iron-regulatory hepcidin by vitamin D. J Am Soc Nephrol.

[30] Smith EM (2017). High-dose vitamin D3 reduces circulating hepcidin concentrations: A pilot, randomized, double-blind, placebo-controlled trial in healthy adults. Clin Nutr.

[31] Zughaier SM, Alvarez JA, Sloan JH, Konrad RJ, Tangpricha V (2014). The role of vitamin D in regulating the iron-hepcidin-ferroportin axis in monocytes. J Clin Transl Endocrinol.

[32] Moran-Lev H (2019). Vitamin D Decreases Hepcidin and Inflammatory Markers in Newly Diagnosed Inflammatory Bowel Disease Paediatric Patients: A Prospective Study. J Crohns Colitis.

[33] Syed S (2017). Vitamin D Status Is Associated with Hepcidin and Hemoglobin Concentrations in Children with Inflammatory Bowel Disease. Inflamm Bowel Dis.

[34] De la Cruz-Gongora, V., Salinas-Rodriguez, A., Villalpando, S. & Flores-Aldana, M. Serum Retinol but Not 25(OH)D Status Is Associated With Serum Hepcidin Levels in Older Mexican Adults. *Nutrients* 11 (2019). 10.3390/nu11050988 PMC6566922 31052280

[35] Smith EM (2015). Vitamin D deficiency is associated with anaemia among African Americans in a US cohort. Br J Nutr.

[36] Sangkhae V, Nemeth E (2017). Regulation of the Iron Homeostatic Hormone Hepcidin. Adv Nutr.

[37] Arnett FC (1988). The American Rheumatism Association 1987 revised criteria for the classification of rheumatoid arthritis. Arthritis Rheum.

[38] Aletaha D (2010). 2010 Rheumatoid arthritis classification criteria: an American College of Rheumatology/European League Against Rheumatism collaborative initiative. Arthritis Rheum.

[39] Matsuo S (2009). Revised equations for estimated GFR from serum creatinine in Japan. Am J Kidney Dis.

[40] Payne RB, Little AJ, Williams RB, Milner JR (1973). Interpretation of serum calcium in patients with abnormal serum proteins. Br Med J.

[41] Yamazaki Y (2002). Increased circulatory level of biologically active full-length FGF-23 in patients with hypophosphatemic rickets/osteomalacia. J Clin Endocrinol Metab.

