# Supplementary material for: Serum hepcidin level, iron metabolism and osteoporosis in patients with rheumatoid arthritis
Source: Sci Rep. 2020 Jun 18;10:9882. doi: 10.1038/s41598-020-66945-3 (PMC7303107; doi:10.1038/s41598-020-66945-3)
Supplement: Supplementary file 1 — Supplementary information. [file 41598_2020_66945_MOESM1_ESM.doc]

**SUPPLEMENTARY TABLES**

**Serum hepcidin level, iron metabolism and osteoporosis in patients with rheumatoid arthritis**

*Scientific Reports*

Hiroe Sato^1,2^, Chinatsu Takai^3^**,** Junichiro James Kazama^4^, Ayako Wakamatsu^2^, Eriko Hasegawa^2^, Daisuke Kobayashi^2^, Naoki Kondo^5^, Takeshi Nakatsue^2^, Asami Abe^3^, Satoshi Ito^3^, Hajime Ishikawa^3^, Takeshi Kuroda^1^, Yoshiki Suzuki^1^, Ichiei Narita^2^

**Correspondence to:**

Hiroe Sato, M.D., Ph.D.

Niigata University Health Administration Center, 2-8050 Ikarashi, Nishi-ku, Niigata City, 950-2181, JAPAN

TEL +81-252-62-6244 FAX +81-252-62-7517

E-mail: [hiroes@med.niigata-u.ac.jp](mailto:hiroes@med.niigata-u.ac.jp)

Table S1. Correlations between serum hepcidin level and other parameters with stratification according to disease activity or bone mineral density

|  |  |  | Serum iron | | Serum ferritin | | UIBC | |
| --- | --- | --- | --- | --- | --- | --- | --- | --- |
|  |  | n | r | p | r | p | r | p |
| DAS28-CRP | Remission (<2.3) | 164 | **0.454** | **<0.001** | **0.812** | **<0.001** | **-0.741** | **<0.001** |
|  | Low disease activity (2.3≤, <2.7) | 38 | **0.455** | **0.004** | **0.674** | **<0.001** | **-0.646** | **<0.001** |
|  | Moderate to high disease activity (2.7≤) | 60 | 0.058 | 0.662 | **0.882** | **<0.001** | **-0.645** | **<0.001** |
| CDAI | Remission (≤2.8) | 93 | **0.518** | **<0.001** | **0.830** | **<0.001** | **-0.801** | **<0.001** |
|  | Low disease activity (2.8<, ≤10) | 123 | **0.242** | **0.007** | **0.813** | **<0.001** | **-0.652** | **<0.001** |
|  | Moderate to high disease activity (10<) | 46 | 0.177 | 0.239 | **0.821** | **<0.001** | **-0.663** | **<0.001** |
| Femoral T score | Osteoporosis (≤-2.5) | 49 | **0.301** | **0.035** | **0.857** | **<0.001** | **-0.701** | **<0.001** |
|  | Osteopenia (-2.5<, <-1.0) | 150 | **0.375** | **<0.001** | **0.791** | **<0.001** | **-0.704** | **<0.001** |
|  | Normal (-1.0≤) | 61 | 0.144 | 0.269 | **0.787** | **<0.001** | **-0.625** | **<0.001** |
| Lumbar T score | Osteoporosis (≤-2.5) | 58 | 0.128 | 0.338 | **0.834** | **<0.001** | **-0.709** | **<0.001** |
|  | Osteopenia (-2.5<, <-1.0) | 96 | **0.357** | **<0.001** | **0.815** | **<0.001** | **-0.739** | **<0.001** |
|  | Normal (-1.0≤) | 108 | **0.378** | **<0.001** | **0.805** | **<0.001** | **-0.671** | **<0.001** |

DAS28, disease activity scores in 28 joints; CDAI, clinical disease activity index

Table S2. Correlations between serum iron, ferritin and hepcidin levels and osteoporosis-related factors with stratification according to disease activity

|  |  | Remission  (CDAI ≤2.8) | | | Low disease activity  (CDAI 2.8<, ≤10) | | | Moderate to high disease activity (CDAI 10<) | | |
| --- | --- | --- | --- | --- | --- | --- | --- | --- | --- | --- |
|  |  | n=93 | | | n=123 | | | n=46 | | |
|  |  | Fe | Ferritin | log(hep+1) | Fe | Ferritin | log(hep+1) | Fe | Ferritin | log(hep+1) |
| Femoral T score | r | **0.318** | 0.050 | 0.043 | 0.171 | 0.060 | -0.022 | 0.175 | -0.165 | -0.154 |
|  | p | **0.002** | 0.636 | 0.683 | 0.061 | 0.516 | 0.811 | 0.246 | 0.272 | 0.308 |
| Femoral Z score | r | 0.174 | 0.050 | 0.026 | 0.104 | 0.163 | 0.139 | 0.151 | -0.039 | -0.031 |
|  | p | 0.096 | 0.634 | 0.802 | 0.257 | 0.074 | 0.129 | 0.318 | 0.799 | 0.838 |
| Lumbar T score | r | 0.154 | 0.053 | -0.003 | -0.028 | -0.014 | -0.150 | 0.086 | -0.215 | -0.192 |
|  | p | 0.141 | 0.611 | 0.979 | 0.758 | 0.882 | 0.097 | 0.568 | 0.151 | 0.201 |
| Lumbar Z score | r | 0.048 | 0.077 | 0.007 | -0.077 | 0.040 | -0.052 | 0.022 | -0.147 | -0.133 |
|  | p | 0.649 | 0.464 | 0.950 | 0.398 | 0.664 | 0.570 | 0.885 | 0.331 | 0.377 |
| Bone alkaline phosphatase | r | -0.105 | -0.029 | 0.010 | -0.056 | 0.081 | 0.096 | 0.080 | -0.167 | -0.283 |
|  | p | 0.315 | 0.779 | 0.921 | 0.537 | 0.372 | 0.292 | 0.596 | 0.266 | 0.056 |
| TRACP-5b | r | -0.082 | -0.039 | 0.012 | -0.137 | 0.071 | 0.071 | -0.202 | -0.123 | -0.288 |
|  | p | 0.436 | 0.709 | 0.906 | 0.132 | 0.435 | 0.438 | 0.179 | 0.417 | 0.052 |
| 25(OH)D | r | 0.147 | **0.356** | **0.342** | 0.082 | **0.293** | **0.204** | -0.235 | -0.069 | -0.047 |
|  | p | 0.159 | **<0.001** | **0.001** | 0.366 | **0.001** | **0.023** | 0.116 | 0.649 | 0.759 |
| FGF-23 | r | 0.008 | 0.076 | -0.027 | -0.007 | 0.025 | -0.022 | 0.112 | -0.060 | -0.002 |
|  | p | 0.943 | 0.469 | 0.796 | 0.935 | 0.787 | 0.805 | 0.457 | 0.690 | 0.990 |
| MMP-3 | r | -0.082 | -0.023 | -0.083 | **-0.236** | -0.005 | -0.049 | -0.165 | 0.262 | 0.156 |
|  | p | 0.436 | 0.825 | 0.429 | **0.008** | 0.954 | 0.589 | 0.274 | 0.078 | 0.301 |
| Annual change of femoral T score* | r | 0.064 | -0.182 | -0.011 | 0.077 | -0.129 | -0.086 | 0.049 | 0.065 | 0.061 |
|  | p | 0.579 | 0.113 | 0.926 | 0.418 | 0.170 | 0.363 | 0.762 | 0.689 | 0.710 |
| Annual change of lumbar T score* | r | 0.166 | 0.176 | **0.293** | -0.001 | 0.108 | 0.084 | -0.063 | 0.192 | 0.051 |
|  | p | 0.150 | 0.127 | **0.010** | 0.994 | 0.254 | 0.377 | 0.698 | 0.236 | 0.754 |

TRACP-5b, tartrate-resistant acid phosphatase-5b; 25(OH)D, 25-hydroxy vitamin D; FGF23, fibroblast growth factor 23; MMP-3, matrix metalloprotease 3.

*Annual change of T score was evaluated in 231 (remission, 77; low disease activity, 114; moderate to high disease activity, 40) patients and the mean observational period was 2.0±0.33 years (0.63 – 3.25).

Table S3. Distribution of factors according to quartiles of serum hepcidin level

|  |  | Q1 |  |  |  | Q2 |  | |  |  | Q3 |  |  |  | Q4 |  |  | *P* |
| --- | --- | --- | --- | --- | --- | --- | --- | --- | --- | --- | --- | --- | --- | --- | --- | --- | --- | --- |
| Hepcidin, ng/mL | (0 | – | 1.3) |  | (1.4 | – | 6.6) | |  | (6.7 | – | 19.5) |  | (20.0 | – | 102.3) |  |  |
|  | 0.5 | ± | 0.4 |  | 3.5 | ± | 1.4 | |  | 11.7 | ± | 3.8 |  | 41.7 | ± | 22.0 |  |  |
| *n* | 66 | | |  | 66 | | | |  | 65 | | |  | 66 | | |  |  |
| Age, years | 67.0 | ± | 12.7 |  | 65.2 | ± | | 13.3 |  | 68.2 | ± | 9.4 |  | 69.4 | ± | 9.5 |  | 0.230 |
| Female, *n* (%) | 50 |  | (75.8) |  | 51 |  | | (78.5) |  | 54 |  | (81.8) |  | 48 |  | (73.8) |  | 0.717 |
| Body mass index, kg/m^2^ | 21.9 | ± | 3.7 |  | 21.8 | ± | | 3.7 |  | 22.2 | ± | 2.9 |  | 21.5 | ± | 3.3 |  | 0.706 |
| Dissease duration, years | 14.5 | ± | 10.3 |  | 13.9 | ± | | 11.3 |  | 12.2 | ± | 9.2 |  | 13.9 | ± | 11.5 |  | 0.342 |
| Rheumatoid factor, U/mL | 153.9 | ± | 335.4 |  | 69.4 | ± | | 133.7 |  | 140.6 | ± | 285.8 |  | 119.7 | ± | 222.8 |  | 0.936 |
| **ESR, mm/h** | **16.5** | **±** | **11.7** |  | **16.6** | **±** | | **16.1** |  | **17.5** | **±** | **13.9** |  | **27.2** | **±** | **24.7** |  | **0.039** |
| **CRP, mg/dL** | **0.2** | **±** | **0.4** |  | **0.1** | **±** | | **0.2** |  | **0.3** | **±** | **0.5** |  | **0.8** | **±** | **1.4** |  | **< 0.001** |
| MMP-3, mg/mL | 130.9 | ± | 102.0 |  | 100.0 | ± | | 56.4 |  | 113.9 | ± | 90.0 |  | 144.9 | ± | 132.6 |  | 0.895 |
| DAS28-ESR | 2.6 | ± | 0.9 |  | 2.6 | ± | | 1.1 |  | 2.4 | ± | 0.7 |  | 2.9 | ± | 1.2 |  | 0.203 |
| DAS28-CRP | 2.0 | ± | 0.9 |  | 2.2 | ± | | 0.9 |  | 1.9 | ± | 0.6 |  | 2.7 | ± | 2.5 |  | 0.070 |
| SDAI | 6.1 | ± | 6.7 |  | 6.8 | ± | | 6.4 |  | 4.3 | ± | 3.1 |  | 7.9 | ± | 7.0 |  | 0.227 |
| CDAI | 5.8 | ± | 6.7 |  | 6.7 | ± | | 6.3 |  | 4.1 | ± | 3.1 |  | 7.1 | ± | 6.4 |  | 0.422 |
| HAQ-DI | 0.6 | ± | 0.8 |  | 0.5 | ± | | 0.7 |  | 0.4 | ± | 0.6 |  | 0.6 | ± | 0.8 |  | 0.226 |
| PSL use, *n* (%) | 57 |  | (86.4) |  | 46 |  | | (70.8) |  | 44 |  | (66.7) |  | 48 |  | (73.8) |  | 0.058 |
| **Daily PSL dose, mg/day** | **3.9** | **±** | **3.8** |  | **2.7** | **±** | | **3.1** |  | **2.3** | **±** | **2.5** |  | **3.1** | **±** | **4.4** |  | **0.016** |
| MTX use, *n* (%) | 36 |  | (54.5) |  | 36 |  | | (55.4) |  | 32 |  | (48.5) |  | 34 |  | (52.3) |  | 0.861 |
| Weekly MTX dose, mg/week | 4.6 | ± | 4.8 |  | 4.2 | ± | | 4.1 |  | 3.4 | ± | 3.9 |  | 3.7 | ± | 4.2 |  | 0.198 |
| **bDMARD use, *n* (%)** | **30** |  | **(45.5)** |  | **27** |  | | **(41.5)** |  | **14** |  | **(21.2)** |  | **17** |  | **(26.2)** |  | **0.007** |
| Tocilizumab, *n* (%) | 6 |  | (9.1) |  | 9 |  | | (13.8) |  | 4 |  | (6.1) |  | 2 |  | (3.1) |  | 0.133 |
| Serum adjusted Ca, mg/dL | 9.4 | ± | 0.4 |  | 9.7 | ± | | 1.3 |  | 9.5 | ± | 0.4 |  | 9.6 | ± | 0.5 |  | 0.149 |
| Serum phosphate, mg/dL | 3.3 | ± | 0.6 |  | 3.3 | ± | | 0.5 |  | 3.4 | ± | 0.6 |  | 3.3 | ± | 0.5 |  | 0.382 |
| eGFR, mL/min/1.73 m^2^ | 72.6 | ± | 19.4 |  | 76.9 | ± | | 20.7 |  | 70.1 | ± | 17.7 |  | 73.5 | ± | 20.4 |  | 0.875 |
| Red blood cell, 10^4^/mL | 421.8 | ± | 45.0 |  | 413.7 | ± | | 55.1 |  | 411.7 | ± | 43.3 |  | 404.4 | ± | 53.1 |  | 0.087 |
| **Hemoglobin, g/dL** | **12.0** | **±** | **1.4** |  | **12.8** | **±** | | **1.5** |  | **12.7** | **±** | **1.1** |  | **12.5** | **±** | **1.5** |  | **0.047** |
| Hemoglobin <9 g/dL, *n*(%) | 1 |  | (1.5) |  | 0 |  | | (0.0) |  | 0 |  | (0.0) |  | 0 |  | (0.0) |  | N.D. |
| Hematocrit,% | 36.6 | ± | 3.8 |  | 38.2 | ± | | 4.2 |  | 38.0 | ± | 3.2 |  | 37.3 | ± | 4.2 |  | 0.307 |
| **Platelet count, 10^4^/mL** | **23.8** | **±** | **5.1** |  | **21.9** | **±** | | **6.4** |  | **20.6** | **±** | **5.1** |  | **21.9** | **±** | **7.0** |  | **0.017** |
| **Serum ferritin, ng/mL** | **16.4** | **±** | **12.7** |  | **38.1** | **±** | | **24.0** |  | **66.1** | **±** | **43.7** |  | **130.0** | **±** | **79.8** |  | **< 0.001** |
| **Serum iron, mg/dL** | **52.2** | **±** | **25.8** |  | **79.2** | **±** | | **35.4** |  | **79.1** | **±** | **26.0** |  | **81.8** | **±** | **40.4** |  | **< 0.001** |
| **UIBC, mg/dL** | **319.1** | **±** | **52.3** |  | **251.1** | **±** | | **50.4** |  | **220.1** | **±** | **46.4** |  | **191.5** | **±** | **49.1** |  | **< 0.001** |
| Femoral T score | –1.5 | ± | 1.1 |  | –1.5 | ± | | 1.1 |  | –1.5 | ± | 1.0 |  | –1.7 | ± | 1.0 |  | 0.508 |
| Femoral Z score | 0.3 | ± | 0.9 |  | 0.3 | ± | | 1.1 |  | 0.5 | ± | 0.9 |  | 0.3 | ± | 1.0 |  | 0.580 |
| Lumbar T score | –1.1 | ± | 1.5 |  | –1.2 | ± | | 1.4 |  | –1.0 | ± | 1.3 |  | –1.6 | ± | 1.7 |  | 0.104 |
| Lumbar Z score | 0.5 | ± | 1.4 |  | 0.4 | ± | | 1.4 |  | 0.8 | ± | 1.4 |  | 0.2 | ± | 1.6 |  | 0.405 |
| Bone alkaline phosphatase, mg/L | 15.7 | ± | 21.1 |  | 13.0 | ± | | 5.6 |  | 13.0 | ± | 5.4 |  | 15.9 | ± | 28.8 |  | 0.893 |
| TRACP-5b, mU/dL | 332.9 | ± | 223.8 |  | 314.9 | ± | | 184.1 |  | 327.4 | ± | 182.4 |  | 301.1 | ± | 203.8 |  | 0.601 |
| **25(OH)D, ng/mL** | **14.8** | **±** | **6.7** |  | **15.9** | **±** | | **5.6** |  | **17.3** | **±** | **6.5** |  | **18.1** | **±** | **8.1** |  | **0.002** |
| FGF23, pg/mL | 57.5 | ± | 23.8 |  | 60.6 | ± | | 35.4 |  | 59.7 | ± | 21.6 |  | 59.1 | ± | 46.0 |  | 0.801 |
| Bisphosphonate or denosumab use, *n* (%) | 45 |  | (68.2) |  | 44 |  | | (67.7) |  | 39 |  | (59.1) |  | 37 |  | (56.9) |  | 0.418 |
| Teriparatide use, *n* (%) | 2 |  | (3.1) |  | 4 |  | | (6.2) |  | 1 |  | (1.5) |  | 2 |  | (3.1) |  | 0.527 |
| Calcium preparation use, *n* (%) | 2 |  | (3.0) |  | 4 |  | | (6.2) |  | 2 |  | (3.0) |  | 3 |  | (4.6) |  | 0.779 |
| Active form of vitamin D preparation, *n* (%) | 7 |  | (10.6) |  | 11 |  | | (16.9) |  | 7 |  | (10.6) |  | 12 |  | (18.5) |  | 0.428 |
| Vitamin K2 use, *n* (%) | 3 |  | (4.5) |  | 3 |  | | (4.6) |  | 1 |  | (1.5) |  | 1 |  | (1.5) |  | 0.559 |
| Iron agent use, *n* (%) | 0 |  | (0.0) |  | 2 |  | | (3.1) |  | 2 |  | (3.1) |  | 5 |  | (7.7) |  | 0.114 |
| Annual change of femoral T score* | -0.010 | ± | 0.187 |  | 0.007 | ± | | 0.130 |  | -0.015 | ± | 0.140 |  | -0.179 | ± | 0.177 |  | 0.610 |
| **Annual change of lumbar T score*** | **0.060** | **±** | **0.236** |  | **0.105** | **±** | | **0.159** |  | **0.101** | **±** | **0.177** |  | **0.149** | **±** | **0.223** |  | **0.026** |

DAS28, disease activity scores in 28 joints; ESR, erythrocyte sedimentation rate; CRP, C-reactive protein; MMP-3, matrix metalloprotease 3; SDAI, simplified disease activity index; CDAI, clinical disease activity index; HAQ-DI, health assessment questionnaire without disability index; DMARDs, disease-modifying antirheumatic drugs; eGFR, estimated glomerular filtration rate; UIBC, unsaturated iron binding capacity; TRACP-5b, tartrate-resistant acid phosphatase-5b; 25(OH)D, 25-hydroxy vitamin D; FGF23, fibroblast growth factor 23; N.D., not determined.

*Annual change of T score was evaluated 231 patients (Q1, n= 61; Q2, n= 58; Q3, n=58; Q4, n=54).

Table S4. Distribution of factors according to quartiles of serum 25(OH)D level

|  |  | Q1 |  |  |  | Q2 |  |  |  | Q3 |  |  |  | Q4 |  |  | *P* |
| --- | --- | --- | --- | --- | --- | --- | --- | --- | --- | --- | --- | --- | --- | --- | --- | --- | --- |
| 25(OH)D, ng/mL | (3.5 | – | 11.6) |  | (11.7 | – | 15.6) |  | (15.8 | – | 20.6) |  | (20.7 | – | 50.3) |  |  |
|  | 8.9 | ± | 1.8 |  | 13.6 | ± | 1.1 |  | 18.0 | ± | 1.4 |  | 25.9 | ± | 5.4 |  |  |
| *n* | 66 | | |  | 65 | | |  | 68 | | |  | 63 | | |  |  |
| **Age, years** | **65.0** | **±** | **12.4** |  | **66.4** | **±** | **12.2** |  | **68.0** | **±** | **11.3** |  | **70.6** | **±** | **8.9** |  | **0.004** |
| Female, *n* (%) | 56 |  | (84.8) |  | 51 |  | (78.5) |  | 52 |  | (76.5) |  | 44 |  | (69.8) |  | 0.237 |
| Body mass index, kg/m^2^ | 21.6 | ± | 3.7 |  | 21.9 | ± | 3.0 |  | 21.7 | ± | 3.6 |  | 22.3 | ± | 3.5 |  | 0.359 |
| Disease duration, years | 13.9 | ± | 10.0 |  | 14.3 | ± | 12.1 |  | 11.8 | ± | 9.1 |  | 14.6 | ± | 11.0 |  | 0.868 |
| Rheumatoid factor, U/mL | 149.1 | ± | 269.6 |  | 136.2 | ± | 311.7 |  | 76.4 | ± | 205.9 |  | 123.3 | ± | 225.6 |  | 0.628 |
| ESR, mm/h | 17.3 | ± | 15.9 |  | 19.5 | ± | 16.2 |  | 16.9 | ± | 14.2 |  | 24.3 | ± | 23.2 |  | 0.144 |
| CRP, mg/dL | 0.4 | ± | 0.8 |  | 0.3 | ± | 0.6 |  | 0.3 | ± | 0.8 |  | 0.5 | ± | 1.0 |  | 0.774 |
| MMP-3, mg/mL | 105.9 | ± | 69.4 |  | 127.4 | ± | 95.1 |  | 124.0 | ± | 103.3 |  | 133.0 | ± | 125.4 |  | 0.587 |
| DAS28-ESR | 2.6 | ± | 1.0 |  | 2.7 | ± | 1.0 |  | 2.5 | ± | 1.0 |  | 2.8 | ± | 1.0 |  | 0.331 |
| DAS28-CRP | 2.1 | ± | 0.9 |  | 2.2 | ± | 0.9 |  | 2.3 | ± | 2.4 |  | 2.2 | ± | 0.9 |  | 0.726 |
| SDAI | 6.5 | ± | 6.9 |  | 7.3 | ± | 7.1 |  | 5.6 | ± | 5.3 |  | 5.9 | ± | 4.8 |  | 0.787 |
| CDAI | 6.1 | ± | 6.6 |  | 6.9 | ± | 7.0 |  | 5.3 | ± | 5.0 |  | 5.4 | ± | 4.6 |  | 0.676 |
| HAQ-DI | 0.6 | ± | 0.9 |  | 0.6 | ± | 0.7 |  | 0.4 | ± | 0.6 |  | 0.5 | ± | 0.6 |  | 0.184 |
| PSL use, *n* (%) | 48 |  | (72.7) |  | 51 |  | (78.5) |  | 50 |  | (73.5) |  | 46 |  | (73.0) |  | 0.861 |
| Daily PSL dose, mg/day | 3.0 | ± | 3.7 |  | 3.5 | ± | 4.3 |  | 3.1 | ± | 3.5 |  | 2.4 | ± | 2.2 |  | 0.539 |
| MTX use, *n*(%) | 64 |  | (51.5) |  | 33 |  | (50.8) |  | 38 |  | (55.9) |  | 33 |  | (52.4) |  | 0.938 |
| Weekly MTX dose, mg/week | 3.9 | ± | 4.2 |  | 4.2 | ± | 4.7 |  | 4.2 | ± | 4.2 |  | 3.6 | ± | 4.0 |  | 0.727 |
| bDMARD use, *n* (%) | 25 |  | (37.9) |  | 23 |  | (35.4) |  | 22 |  | (32.4) |  | 18 |  | (28.6) |  | 0.706 |
| Tocilizumab, *n*(%) | 5 |  | (7.6) |  | 8 |  | (12.3) |  | 7 |  | (10.3) |  | 1 |  | (1.6) |  | 0.130 |
| **Serum adjusted Ca, mg/dL** | **9.5** | **±** | **0.4** |  | **9.5** | **±** | **0.4** |  | **9.7** | **±** | **1.3** |  | **9.6** | **±** | **0.4** |  | **0.019** |
| Serum phosphate, mg/dL | 3.3 | ± | 0.6 |  | 3.3 | ± | 0.5 |  | 3.4 | ± | 0.6 |  | 3.3 | ± | 0.6 |  | 0.641 |
| **eGFR, mL/min/1.73 m^2^** | **77.0** | **±** | **17.7** |  | **74.3** | **±** | **19.5** |  | **74.2** | **±** | **20.9** |  | **67.4** | **±** | **19.2** |  | **0.014** |
| Red blood cell, 10^4^/mL | 419.0 | ± | 45.7 |  | 405.0 | ± | 51.5 |  | 412.4 | ± | 54.2 |  | 415.5 | ± | 45.5 |  | 0.671 |
| Hemoglobin, g/dL | 12.5 | ± | 1.4 |  | 12.3 | ± | 1.4 |  | 12.6 | ± | 1.5 |  | 12.5 | ± | 1.3 |  | 0.611 |
| Hematocrit,% | 37.8 | ± | 4.0 |  | 36.9 | ± | 3.9 |  | 37.8 | ± | 4.2 |  | 37.6 | ± | 3.5 |  | 0.858 |
| Platelet count, 10^4^/mL | 23.3 | ± | 6.8 |  | 21.5 | ± | 6.1 |  | 21.0 | ± | 4.8 |  | 22.5 | ± | 6.1 |  | 0.217 |
| **Serum ferritin, ng/mL** | **44.2** | **±** | **46.1** |  | **54.6** | **±** | **53.1** |  | **74.2** | **±** | **72.8** |  | **77.1** | **±** | **73.2** |  | **< 0.001** |
| Serum iron, mg/dL | 68.1 | ± | 35.2 |  | 79.3 | ± | 38.6 |  | 73.2 | ± | 31.2 |  | 71.5 | ± | 32.4 |  | 0.540 |
| UIBC, mg/dL | 262.0 | ± | 70.3 |  | 242.5 | ± | 76.2 |  | 237.9 | ± | 61.8 |  | 240.1 | ± | 63.9 |  | 0.081 |
| **Serum hepcidin, ng/mL** | **9.6** | **±** | **13.7** |  | **13.4** | **±** | **20.2** |  | **15.0** | **±** | **19.1** |  | **19.3** | **±** | **24.0** |  | **0.004** |
| **Log 10 (hepcidin + 1)** | **0.7** | **±** | **0.5** |  | **0.8** | **±** | **0.5** |  | **0.9** | **±** | **0.5** |  | **1.0** | **±** | **0.6** |  | **0.004** |
| Femoral T score | –1.6 | ± | 1.1 |  | –1.5 | ± | 1.1 |  | –1.7 | ± | 1.0 |  | –1.4 | ± | 1.1 |  | 0.781 |
| **Femoral Z score** | **0.2** | **±** | **1.0** |  | **0.3** | **±** | **1.0** |  | **0.3** | **±** | **1.0** |  | **0.6** | **±** | **1.0** |  | **0.049** |
| Lumbar T score | –1.0 | ± | 1.4 |  | –1.2 | ± | 1.4 |  | –1.4 | ± | 1.5 |  | –1.3 | ± | 1.7 |  | 0.074 |
| Lumbar Z score | 0.5 | ± | 1.4 |  | 0.4 | ± | 1.4 |  | 0.4 | ± | 1.4 |  | 0.6 | ± | 1.5 |  | 0.876 |
| Bone alkaline phosphatase, mg/L | 13.2 | ± | 4.8 |  | 13.4 | ± | 6.0 |  | 12.0 | ± | 5.0 |  | 19.3 | ± | 35.6 |  | 0.660 |
| TRACP-5b, mU/dL | 343.9 | ± | 221.6 |  | 338.8 | ± | 220.7 |  | 281.9 | ± | 164.0 |  | 313.3 | ± | 180.1 |  | 0.237 |
| **FGF23, pg/mL** | **51.0** | **±** | **23.0** |  | **61.4** | **±** | **33.6** |  | **56.9** | **±** | **24.3** |  | **68.3** | **±** | **45.2** |  | **0.002** |
| Bisphosphonate or denosumab use, *n* (%) | 40 |  | (60.6) |  | 40 |  | (61.5) |  | 44 |  | (64.7) |  | 41 |  | (65.1) |  | 0.935 |
| Teriparatide use, *n* (%) | 1 |  | (1.5) |  | 5 |  | (7.7) |  | 1 |  | (1.5) |  | 2 |  | (3.2) |  | 0.169 |
| Calcium preparation use, *n* (%) | 1 |  | (1.5) |  | 3 |  | (4.6) |  | 4 |  | (5.9) |  | 3 |  | (4.8) |  | 0.628 |
| Active form of vitamin D preparation use, *n* (%) | 9 |  | (13.6) |  | 11 |  | (16.9) |  | 9 |  | (13.2) |  | 8 |  | (12.8) |  | 0.900 |
| Vitamin K2 use, *n* (%) | 2 |  | (3.0) |  | 4 |  | (6.2) |  | 2 |  | (2.9) |  | 0 |  | (0.0) |  | 0.251 |
| Iron agent use, *n* (%) | 2 |  | (3.0) |  | 3 |  | (4.6) |  | 4 |  | (5.9) |  | 0 |  | (0.0) |  | 0.287 |
| Annual change of femoral T score* | 0.008 | ± | 0.191 |  | -0.026 | ± | 0.156 |  | -0.019 | ± | 0.137 |  | 0.003 | ± | 0.154 |  | 0.319 |
| **Annual change of lumbar T score*** | **0.085** | **±** | **0.239** |  | **0.057** | **±** | **0.194** |  | **0.116** | **±** | **0.162** |  | **0.155** | **±** | **0.205** |  | **0.011** |

DAS28, disease activity scores in 28 joints; ESR, erythrocyte sedimentation rate; CRP, C-reactive protein; MMP-3, matrix metalloprotease 3; SDAI, simplified disease activity index; CDAI, clinical disease activity index; HAQ-DI, health assessment questionnaire without disability index; DMARDs, disease-modifying antirheumatic drugs; eGFR, estimated glomerular filtration rate; UIBC, unsaturated iron binding capacity; TRACP-5b, tartrate-resistant acid phosphatase-5b; 25(OH)D, 25-hydroxy vitamin D; FGF23, fibroblast growth factor 23.

*Annual change of T score was evaluated 231 patients (Q1, n= 55; Q2, n= 61; Q3, n=61; Q4, n=54).

Table S5. Distribution of factors according to quartiles of serum 25(OH)D level in patients without active form of vitamin D preparation treatment

|  |  |  | Q1 |  |  |  | Q2 |  |  |  | Q3 |  |  |  | Q4 |  |  | *P* |
| --- | --- | --- | --- | --- | --- | --- | --- | --- | --- | --- | --- | --- | --- | --- | --- | --- | --- | --- |
| 25OHVD, ng/mL |  | (3.5 | – | 11.6) |  | (11.7 | – | 15.6) |  | (15.8 | – | 20.6) |  | (20.7 | – | 50.3) |  |  |
|  |  | 9.0 | ± | 1.9 |  | 13.6 | ± | 1.2 |  | 17.9 | ± | 1.4 |  | 26.1 | ± | 5.5 |  |  |
| *n* |  | 59 | | |  | 54 | | |  | 59 | | |  | 53 | | |  |  |
| **Age, years** |  | **64.3** | **±** | **12.6** |  | **65.6** | **±** | **13.0** |  | **68.5** | **±** | **10.3** |  | **70.6** | **±** | **8.6** |  | **0.002** |
| Female, *n* (%) |  | 50 |  | (84.7) |  | 40 |  | (74.1) |  | 46 |  | (78.0) |  | 38 |  | (71.7) |  | 0.467 |
| Body mass index, kg/m^2^ |  | 21.5 | ± | 3.8 |  | 22.1 | ± | 3.0 |  | 21.9 | ± | 3.5 |  | 22.7 | ± | 3.3 |  | 0.080 |
| Disease duration, years |  | 14.4 | ± | 10.1 |  | 13.5 | ± | 11.8 |  | 11.7 | ± | 9.4 |  | 13.9 | ± | 10.5 |  | 0.569 |
| Rheumatoid factor, U/mL |  | 142.1 | ± | 267.4 |  | 94.1 | ± | 170.1 |  | 69.1 | ± | 187.9 |  | 113.1 | ± | 190.8 |  | 0.723 |
| ESR, mm/h |  | 16.9 | ± | 15.8 |  | 17.6 | ± | 14.5 |  | 16.7 | ± | 14.1 |  | 22.8 | ± | 22.9 |  | 0.280 |
| CRP, mg/dL |  | 0.4 | ± | 0.8 |  | 0.2 | ± | 0.5 |  | 0.3 | ± | 0.8 |  | 0.5 | ± | 1.0 |  | 0.821 |
| MMP-3, mg/mL |  | 101.7 | ± | 68.3 |  | 126.6 | ± | 91.0 |  | 119.4 | ± | 100.5 |  | 131.4 | ± | 129.6 |  | 0.597 |
| DAS28-ESR |  | 2.5 | ± | 1.0 |  | 2.5 | ± | 0.7 |  | 2.5 | ± | 1.0 |  | 2.7 | ± | 0.9 |  | 0.318 |
| DAS28-CRP |  | 2.1 | ± | 0.9 |  | 2.0 | ± | 0.7 |  | 2.3 | ± | 2.6 |  | 2.2 | ± | 0.9 |  | 0.723 |
| SDAI |  | 6.1 | ± | 6.1 |  | 5.6 | ± | 4.9 |  | 5.8 | ± | 5.5 |  | 5.6 | ± | 4.6 |  | 0.955 |
| CDAI |  | 5.7 | ± | 5.6 |  | 5.3 | ± | 4.8 |  | 5.6 | ± | 5.2 |  | 5.1 | ± | 4.4 |  | 0.832 |
| HAQ-DI |  | 0.6 | ± | 0.9 |  | 0.5 | ± | 0.7 |  | 0.3 | ± | 0.6 |  | 0.4 | ± | 0.6 |  | 0.315 |
| PSL use, *n* (%) |  | 41 |  | (71.9) |  | 42 |  | (77.8) |  | 43 |  | (72.9) |  | 41 |  | (74.5) |  | 0.903 |
| Daily PSL dose, mg/day |  | 3.1 | ± | 3.9 |  | 3.7 | ± | 4.6 |  | 3.0 | ± | 3.6 |  | 2.5 | ± | 2.2 |  | 0.605 |
| MTX use, *n* (%) |  | 31 |  | (54.4) |  | 26 |  | (48.1) |  | 31 |  | (52.5) |  | 29 |  | (52.7) |  | 0.926 |
| Weekly MTX dose, mg/week |  | 4.1 | ± | 4.2 |  | 4.3 | ± | 4.8 |  | 3.9 | ± | 4.2 |  | 3.7 | ± | 4.1 |  | 0.727 |
| bDMARD use, *n* (%) |  | 21 |  | (36.8) |  | 20 |  | (37.0) |  | 19 |  | (32.2) |  | 14 |  | (25.5) |  | 0.529 |
| **Tocilizumab, *n* (%)** |  | **3** |  | **(5.3)** |  | **7** |  | **(13.0)** |  | **6** |  | **(10.2)** |  | **0** |  | **(0.0)** |  | **0.043** |
| Serum adjusted Ca, mg/dL |  | 9.5 | ± | 0.4 |  | 9.5 | ± | 0.4 |  | 9.5 | ± | 0.4 |  | 9.6 | ± | 0.4 |  | 0.054 |
| Serum phosphate, mg/dL |  | 3.3 | ± | 0.6 |  | 3.3 | ± | 0.5 |  | 3.4 | ± | 0.6 |  | 3.3 | ± | 0.6 |  | 0.482 |
| Serum creatinine, mg/dL |  | 0.7 | ± | 0.2 |  | 0.7 | ± | 0.2 |  | 0.7 | ± | 0.2 |  | 0.8 | ± | 0.3 |  | 0.055 |
| **eGFR, mL/min/1.73 m^2^** |  | **77.9** | **±** | **18.3** |  | **73.9** | **±** | **19.6** |  | **74.6** | **±** | **20.8** |  | **68.0** | **±** | **18.6** |  | **0.024** |
| WBC, /mL |  | 5845.6 | ± | 2042.7 |  | 5694.8 | ± | 2026.1 |  | 5791.4 | ± | 1671.6 |  | 5830.9 | ± | 1987.2 |  | 0.842 |
| RBC, 10^4^/mL |  | 418.5 | ± | 46.4 |  | 407.2 | ± | 52.4 |  | 410.6 | ± | 52.6 |  | 416.3 | ± | 47.4 |  | 0.854 |
| Hemoglobin, g/dL |  | 12.5 | ± | 1.5 |  | 12.4 | ± | 1.5 |  | 12.5 | ± | 1.4 |  | 12.6 | ± | 1.3 |  | 0.496 |
| Hematocrit, % |  | 37.7 | ± | 4.1 |  | 37.0 | ± | 4.0 |  | 37.6 | ± | 4.1 |  | 37.7 | ± | 3.6 |  | 0.746 |
| Platelet, 10^4^/mL |  | 23.3 | ± | 6.5 |  | 21.7 | ± | 6.0 |  | 21.0 | ± | 5.0 |  | 22.5 | ± | 6.4 |  | 0.157 |
| **Serum ferritin, ng/mL** |  | **45.9** | **±** | **48.9** |  | **47.7** | **±** | **43.1** |  | **72.0** | **±** | **73.4** |  | **73.8** | **±** | **73.7** |  | **0.002** |
| Serum iron, mg/dL |  | 66.8 | ± | 36.3 |  | 81.4 | ± | 38.6 |  | 71.3 | ± | 29.8 |  | 72.4 | ± | 31.8 |  | 0.420 |
| UIBC, mg/dL |  | 262.1 | ± | 71.8 |  | 246.0 | ± | 76.1 |  | 238.7 | ± | 63.2 |  | 237.9 | ± | 64.2 |  | 0.086 |
| **Serum hepcidin, ng/mL** |  | **9.2** | **±** | **12.7** |  | **12.0** | **±** | **17.8** |  | **14.9** | **±** | **19.5** |  | **19.1** | **±** | **25.0** |  | **0.010** |
| **Log 10 (hepcidin + 1)** |  | **0.7** | **±** | **0.5** |  | **0.8** | **±** | **0.5** |  | **0.9** | **±** | **0.5** |  | **1.0** | **±** | **0.6** |  | **0.010** |
| Femoral T score |  | –1.5 | ± | 1.1 |  | –1.4 | ± | 1.0 |  | –1.6 | ± | 0.9 |  | –1.3 | ± | 1.1 |  | 0.738 |
| **Femoral Z score** |  | **0.2** | **±** | **1.0** |  | **0.4** | **±** | **1.0** |  | **0.4** | **±** | **0.9** |  | **0.7** | **±** | **0.9** |  | **0.014** |
| Lumbar T score |  | –0.9 | ± | 1.4 |  | –1.1 | ± | 1.4 |  | –1.3 | ± | 1.5 |  | –1.1 | ± | 1.7 |  | 0.109 |
| Lumbar Z score |  | 0.6 | ± | 1.4 |  | 0.5 | ± | 1.4 |  | 0.5 | ± | 1.4 |  | 0.7 | ± | 1.4 |  | 0.739 |
| Bone alkaline phosphatase, mg/L |  | 13.5 | ± | 4.9 |  | 13.2 | ± | 5.9 |  | 12.2 | ± | 5.2 |  | 20.6 | ± | 38.0 |  | 0.806 |
| TRACP-5b, mU/dL |  | 360.2 | ± | 224.0 |  | 337.8 | ± | 206.9 |  | 292.8 | ± | 166.5 |  | 316.6 | ± | 180.4 |  | 0.141 |
| **FGF23, pg/mL** |  | **50.6** | **±** | **23.2** |  | **57.3** | **±** | **27.2** |  | **55.1** | **±** | **23.3** |  | **62.3** | **±** | **22.0** |  | **0.003** |
| Bisphosphonate or denosumab use, *n* (%) |  | 33 |  | (57.9) |  | 31 |  | (57.4) |  | 37 |  | (62.7) |  | 36 |  | (65.5) |  | 0.788 |
| Teriparatide use, *n* (%) |  | 1 |  | (1.8) |  | 4 |  | (7.4) |  | 1 |  | (1.7) |  | 2 |  | (3.6) |  | 0.330 |
| Calcium preparation use, *n* (%) |  | 0 |  | (0.0) |  | 1 |  | (1.9) |  | 0 |  | (0.0) |  | 1 |  | (1.8) |  | 0.542 |
| Vitamin K2 use, *n* (%) |  | 2 |  | (3.5) |  | 4 |  | (7.4) |  | 2 |  | (3.4) |  | 0 |  | (0.0) |  | 0.224 |
| Iron agent use, *n* (%) |  | 2 |  | (3.5) |  | 2 |  | (3.5) |  | 3 |  | (5.1) |  | 0 |  | (0.0) |  | 0.454 |
| Annual change of femoral T score* |  | 0.015 | ± | 0.199 |  | -0.023 | ± | 0.156 |  | -0.018 | ± | 0.136 |  | -0.004 | ± | 0.157 |  | 0.689 |
| **Annual change of lumbar T score*** |  | **0.057** |  | **0.215** |  | **0.042** |  | **0.201** |  | **0.111** |  | **0.164** |  | **0.153** |  | **0.208** |  | **0.004** |

DAS28, disease activity scores in 28 joints; ESR, erythrocyte sedimentation rate; CRP, C-reactive protein; MMP-3, matrix metalloprotease 3; SDAI, simplified disease activity index; CDAI, clinical disease activity index; HAQ-DI, health assessment questionnaire without disability index; DMARDs, disease-modifying antirheumatic drugs; eGFR, estimated glomerular filtration rate; UIBC, unsaturated iron binding capacity; TRACP-5b, tartrate-resistant acid phosphatase-5b; 25(OH)D, 25-hydroxy vitamin D; FGF23, fibroblast growth factor 23.

*Annual change of T score was evaluated 204 patients (Q1, n= 48; Q2, n= 52; Q3, n=54; Q4, n=50).

Table S6. Distribution of factors according to quartiles of serum FGF23 level

|  |  |  | Q1 |  |  | |  | | Q2 |  | |  | |  | | | Q3 |  |  |  | Q4 | |  |  | | *P* | |
| --- | --- | --- | --- | --- | --- | --- | --- | --- | --- | --- | --- | --- | --- | --- | --- | --- | --- | --- | --- | --- | --- | --- | --- | --- | --- | --- | --- |
| FGF23, pg/mL |  | (0 | – | 40.3) |  | | (40.7 | | – | 54.0) | |  | | (54.1 | | | – | 73.1) |  | (73.2 | – | | 376) |  | |  | |
|  |  | 29.2 | ± | 9.4 |  | | 47.9 | | ± | 4.0 | |  | | 62.8 | | | ± | 5.8 |  | 98.2 | ± | | 41.4 |  | |  | |
| *n* |  | 66 | | |  | | 65 | | | | |  | | 67 | | | | |  | 64 | | | |  | |  | |
| **Age, years** |  | **65.1** | **±** | **10.4** |  | | **66.2** | | **±** | **11.7** | |  | | **66.6** | | | **±** | **11.5** |  | **72.1** | | **±** | **11.0** |  | | **< 0.001** | |
| Female, *n* (%) |  | 57 |  | (86.4) |  | | 51 | |  | (77.3) | |  | | 51 | | |  | (77.3) |  | 44 | |  | (66.7) |  | | 0.117 | |
| **Body mass index, kg/m^2^** |  | **20.9** | **±** | **3.2** |  | | **21.7** | | **±** | **3.9** | |  | | **22.3** | | | **±** | **3.4** |  | **22.5** | | **±** | **3.1** |  | | **0.002** | |
| Disease duration, years |  | 12.9 | **±** | 11.4 |  | | 15.1 | | **±** | 10.3 | |  | | 12.3 | | | **±** | 9.3 |  | 14.3 | | **±** | 11.3 |  | | 0.720 | |
| **Rheumatoid factor, U/mL** |  | **62.1** | **±** | **131.5** |  | | **114.5** | | **±** | **223.2** | |  | | **131.1** | | | **±** | **300.1** |  | **177.8** | | **±** | **320.8** |  | | **0.004** | |
| **ESR, mm/h** |  | **15.1** | **±** | **15.4** |  | | **18.1** | | **±** | **15.4** | |  | | **22.4** | | | **±** | **21.0** |  | **22.0** | | **±** | **18.0** |  | | **0.001** | |
| CRP, mg/dL |  | 0.3 | ± | 0.8 |  | | 0.3 | | ± | 0.6 | |  | | 0.5 | | | ± | 1.1 |  | 0.3 | | ± | 0.6 |  | | 0.088 | |
| **MMP-3, mg/mL** |  | **102.6** | **±** | **77.1** |  | | **108.3** | | **±** | **89.0** | |  | | **132.8** | | | **±** | **124.9** |  | **146.3** | | **±** | **97.7** |  | | **0.001** | |
| **DAS28-ESR** |  | **2.4** | **±** | **1.0** |  | | **2.6** | | **±** | **0.9** | |  | | **2.7** | | | **±** | **0.9** |  | **2.8** | | **±** | **1.1** |  | | **0.004** | |
| DAS28-CRP |  | 2.0 | ± | 0.9 |  | | 2.1 | | ± | 0.8 | |  | | 2.5 | | | ± | 2.5 |  | 2.2 | | ± | 0.9 |  | | 0.147 | |
| SDAI |  | 6.3 | ± | 6.5 |  | | 6.3 | | ± | 5.6 | |  | | 6.1 | | | ± | 6.0 |  | 6.5 | | ± | 6.4 |  | | 0.836 | |
| CDAI |  | 6.0 | ± | 6.1 |  | | 5.9 | | ± | 5.3 | |  | | 5.6 | | | ± | 5.7 |  | 6.2 | | ± | 6.5 |  | | 0.930 | |
| HAQ-DI |  | 0.4 | ± | 0.7 |  | | 0.6 | | ± | 0.7 | |  | | 0.4 | | | ± | 0.6 |  | 0.7 | | ± | 0.8 |  | | 0.097 | |
| **PSL use, *n* (%)** |  | **44** |  | **(66.7)** |  | | **52** | |  | **(78.8)** | |  | | **45** | | |  | **(68.2)** |  | **54** | |  | **(81.8)** |  | | **0.040** | |
| Daily PSL dose, mg/day |  | 3.0 | ± | 4.5 |  | | 3.1 | | ± | 3.5 | |  | | 2.4 | | | ± | 2.5 |  | 3.6 | | ± | 3.3 |  | | 0.070 | |
| **MTX use, *n* (%)** |  | **46** |  | **(69.7)** |  | | **33** | |  | **(50.0)** | |  | | **34** | | |  | **(51.5)** |  | **25** | |  | **(37.9)** |  | | **0.006** | |
| **Weekly MTX dose, mg/week** |  | **5.2** | **±** | **4.1** |  | | **3.5** | | **±** | **3.9** | |  | | **4.5** | | | **±** | **4.8** |  | **2.7** | | **±** | **3.8** |  | | **0.004** | |
| bDMARD use, *n* (%) |  | 21 |  | (31.8) |  | | 24 | |  | (36.4) | |  | | 23 | | |  | (34.8) |  | 20 | |  | (30.3) |  | | 0.899 | |
| Tocilizumab, *n* (%) |  | 7 |  | (10.6) |  | | 4 | |  | (6.1) | |  | | 7 | | |  | (10.6) |  | 3 | |  | (4.5) |  | | 0.493 | |
| **Serum albumin, g/dL** |  | **4.2** | **±** | **0.5** |  | | **4.1** | | **±** | **0.3** | |  | | **4.0** | | | **±** | **0.5** |  | **4.0** | | **±** | **0.4** |  | | **0.013** | |
| **Serum adjusted Ca, mg/dL** |  | **9.4** | **±** | **0.5** |  | | **9.5** | | **±** | **0.4** | |  | | **9.5** | | | **±** | **0.4** |  | **9.8** | | **±** | **1.3** |  | | **0.007** | |
| Serum phosphate, mg/dL |  | 3.3 | ± | 0.6 |  | | 3.3 | | ± | 0.6 | |  | | 3.4 | | | ± | 0.6 |  | 3.4 | | ± | 0.6 |  | | 0.571 | |
| **Serum creatinine, mg/dL** |  | **0.6** | **±** | **0.1** |  | | **0.7** | | **±** | **0.2** | |  | | **0.7** | | | **±** | **0.2** |  | **0.9** | | **±** | **0.3** |  | | **< 0.001** | |
| **eGFR, mL/min/1.73 m^2^** |  | **80.1** | **±** | **15.5** |  | | **77.5** | | **±** | **19.2** | |  | | **75.0** | | | **±** | **17.0** |  | **60.2** | | **±** | **20.5** |  | | **< 0.001** | |
| **White blood cell, 10^4^/mL** |  | **5539.1** | **±** | **1998.2** |  | | **5699.8** | | **±** | **1727.0** | |  | | **5661.4** | | | **±** | **2087.9** |  | **6452.0** | | **±** | **2009.9** |  | | **0.010** | |
| Red blood cell, 10^4^/mL |  | 414.5 | ± | 42.0 |  | | 422.3 | | ± | 41.9 | |  | | 409.8 | | | ± | 51.4 |  | 405.1 | | ± | 59.9 |  | | 0.060 | |
| **Hemoglobin, g/dL** |  | **12.7** | **±** | **1.3** |  | | **12.6** | | **±** | **1.2** | |  | | **12.4** | | | **±** | **1.4** |  | **12.3** | | **±** | **1.6** |  | | **0.020** | |
| **Hematocrit,%** |  | **38.0** | **±** | **3.6** |  | | **37.9** | | **±** | **3.4** | |  | | **37.2** | | | **±** | **3.9** |  | **36.9** | | **±** | **4.6** |  | | **0.020** | |
| Platelet count, 10^4^/mL |  | 21.8 | ± | 6.0 |  | | 22.1 | | ± | 6.4 | |  | | 22.3 | | | ± | 5.4 |  | 22.1 | | ± | 6.3 |  | | 0.929 | |
| Serum ferritin, ng/mL |  | 61.9 | ± | 54.6 |  | | 55.8 | | ± | 62.9 | |  | | 64.5 | | | ± | 67.1 |  | 67.9 | | ± | 69.7 |  | | 0.699 | |
| Serum iron, mg/dL |  | 74.2 | ± | 36.3 |  | | 69.6 | | ± | 34.7 | |  | | 73.3 | | | ± | 34.4 |  | 74.9 | | ± | 33.0 |  | | 0.731 | |
| UIBC, mg/dL |  | 244.3 | ± | 76.1 |  | | 258.1 | | ± | 62.2 | |  | | 239.1 | | | ± | 65.9 |  | 241.1 | | ± | 69.1 |  | | 0.429 | |
| Alkaline phosphate, U/L |  | 245.3 | ± | 97.0 |  | | 252.0 | | ± | 79.0 | |  | | 222.3 | | | ± | 70.7 |  | 233.5 | | ± | 70.1 |  | | 0.255 | |
| Serum hepcidin, ng/mL |  | 15.5 | ± | 19.7 |  | | 12.4 | | ± | 17.4 | |  | | 15.9 | | | ± | 20.4 |  | 13.1 | | ± | 21.4 |  | | 0.693 | |
| log 10 (hepcidin + 1) |  | 0.9 | ± | 0.6 |  | 0.8 | | ± | | | 0.5 | |  | | 0.9 | ± | | 0.6 |  | 0.8 | ± | | 0.5 | |  | | 0.693 |
| Femoral T score |  | –1.6 | ± | 1.0 |  | –1.6 | | ± | | | 0.9 | |  | | –1.4 | ± | | 1.2 |  | –1.6 | ± | | 1.1 | |  | | 0.910 |
| Femoral Z score |  | 0.3 | ± | 1.0 |  | 0.3 | | ± | | | 0.8 | |  | | 0.4 | ± | | 1.1 |  | 0.4 | ± | | 1.0 | |  | | 0.422 |
| Lumbar T score |  | –1.2 | ± | 1.6 |  | –1.2 | | ± | | | 1.5 | |  | | –1.3 | ± | | 1.5 |  | –1.1 | ± | | 1.4 | |  | | 0.399 |
| Lumbar Z score |  | 0.4 | ± | 1.5 |  | 0.5 | | ± | | | 1.4 | |  | | 0.3 | ± | | 1.3 |  | 0.7 | ± | | 1.5 | |  | | 0.101 |
| **Bone alkaline phosphate, mg/L** |  | **13.6** | **±** | **6.1** |  | **13.9** | | **±** | | | **5.5** | |  | | **14.5** | **±** | | **20.7** |  | **15.7** | **±** | | **29.1** | |  | | **0.046** |
| TRACP-5b, mU/dL |  | 314.2 | ± | 214.4 |  | 352.9 | | ± | | | 217.4 | |  | | 296.6 | ± | | 151.3 |  | 313.7 | ± | | 205.2 | |  | | 0.918 |
| **25(OH)D, ng/mL** |  | **14.6** | **±** | **5.4** |  | **15.5** | | **±** | | | **6.4** | |  | | **17.0** | **±** | | **6.1** |  | **19.1** | **±** | | **8.5** | |  | | **< 0.001** |
| Bisphosphonate or denosumab use, *n* (%) |  | 43 |  | (65.2) |  | 39 | |  | | | (59.1) | |  | | 42 |  | | (63.6) |  | 41 |  | | (62.1) | |  | | 0.937 |
| Teriparatide use, *n* (%) |  | 1 |  | (1.5) |  | 3 | |  | | | (4.5) | |  | | 2 |  | | (3.0) |  | 3 |  | | (4.5) | |  | | 0.725 |
| Calcium preparation use, *n*(%) |  | 1 |  | (1.5) |  | 0 | |  | | | (0.0) | |  | | 5 |  | | (7.6) |  | 5 |  | | (7.6) | |  | | 0.048 |
| Active form of vitamin D preparation use, *n* (%) |  | 8 |  | (12.1) |  | 2 | |  | | | (3.0) | |  | | 12 |  | | (18.2) |  | 15 |  | | (22.7) | |  | | 0.007 |
| Vitamin K2 use, *n* (%) |  | 0 |  | (0.0) |  | 3 | |  | | | (4.5) | |  | | 1 |  | | (1.5) |  | 4 |  | | (6.1) | |  | | 0.146 |
| Iron agent use, *n* (%) |  | 3 |  | (4.5) |  | 0 | |  | | | (0.0) | |  | | 4 |  | | (6.0) |  | 2 |  | | (3.1) | |  | | 0.275 |
| Annual change of femoral T score* |  | -0.184 | ± | 0.136 |  | 0.015 | | ± | | | 0.186 | |  | | -0.000 | ± | | 0.174 |  | -0.038 | ± | | 0.129 | |  | | 0.498 |
| Annual change of lumbar T score* |  | 0.088 | ± | 0.172 |  | 0.109 | | ± | | | 0.232 | |  | | 0.095 | ± | | 0.216 |  | 0.120 | ± | | 0.184 | |  | | 0.455 |

DAS28, disease activity scores in 28 joints; ESR, erythrocyte sedimentation rate; CRP, C-reactive protein; MMP-3, matrix metalloprotease 3; SDAI, simplified disease activity index; CDAI, clinical disease activity index; HAQ-DI, health assessment questionnaire without disability index; DMARDs, disease-modifying antirheumatic drugs; eGFR, estimated glomerular filtration rate; UIBC, unsaturated iron binding capacity; TRACP-5b, tartrate-resistant acid phosphatase-5b; 25(OH)D, 25-hydroxy vitamin D; FGF23, fibroblast growth factor 23.

*Annual change of T score was evaluated 231 patients (Q1, n= 59; Q2, n= 63; Q3, n=58; Q4, n=51).
